# New anti-inflammatory guaianes from the Atlantic hydrotherm-derived fungus *Graphostroma* sp. MCCC 3A00421

**DOI:** 10.1038/s41598-017-18841-6

**Published:** 2018-01-11

**Authors:** Siwen Niu, Chun-Lan Xie, Jin-Mei Xia, Zhu-Hua Luo, Zongze Shao, Xian-Wen Yang

**Affiliations:** State Key Laboratory Breeding Base of Marine Genetic Resources, Key Laboratory of Marine Genetic Resources, Fujian Key Laboratory of Marine Genetic Resources, South China Sea Bio-Resource Exploitation and Utilization Collaborative Innovation Center, Third Institute of Oceanography, State Oceanic Administration, 184 Daxue Road, Xiamen, 361005 People’s Republic of China

## Abstract

Nine new guaianes (graphostromanes A**–**I, **1–9**) were isolated from the deep-sea-derived fungus *Graphostroma* sp. MCCC 3A00421, along with four known ones (**10–13**). The relative configurations were established mainly by detailed analysis of the NMR and HRESIMS data, while the absolute configurations were assigned using the X-ray crystallography and modified Mosher’s method. All isolates were evaluated for their inhibitory effects against lipopolysaccharide (LPS)-induced nitric oxide (NO) production in RAW264.7 macrophages. Graphostromanes F (**6**) showed remarkable inhibitory effect with an IC_50_ value of 14.2 *μ*M, which was even stronger than that of aminoguanidine, a positive control with an IC_50_ value of 23.4 *μ*M.

## Introduction

**Figure 1:**
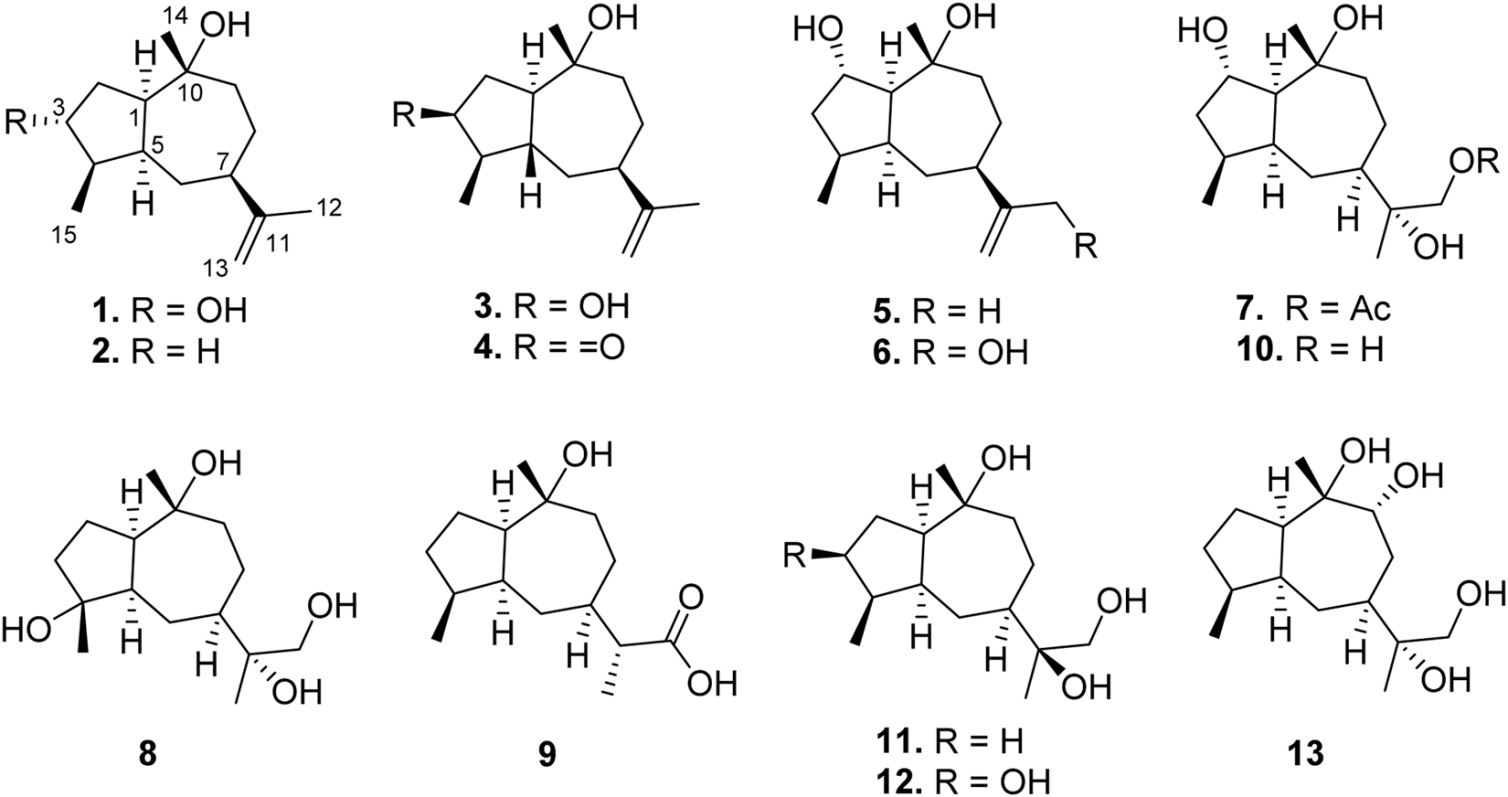
Chemical structures of **1**–**13**.

Guaianes are sesquiterpenoids bearing a bicyclo[5.3.0]-decane skeleton with enormously structural diversities, including nor-guaianes^1^, guaiane alkaloids^2,3^, guaiane dimers^4,5^, *etc*. They possessed a variety of intriguingly biological activities, such as antioxidant^2^, antimalarial^6,7^, antinociceptive^7^, anti-emetic^8^, antitumor^9–11^, anti-inflammatory^12,13^, and antibacteria^14,15^. Guaianes occur mainly in terrestrial plants^16^. Very rarely they were found from terrestrial microorganisms and corals^17–19^. However, they have never been isolated from marine microbes. In our continuing investigation on the deep-sea-derived *Graphostroma* sp. MCCC 3A00421^20^, nine new (**1**–**9**) and four known (**10**–**13**) guaianes were obtained (Fig. 1). Herein, we report the isolation, structure elucidation, and anti-inflammatory activities of these compounds.

## Results and Discussion

**Figure 2:**
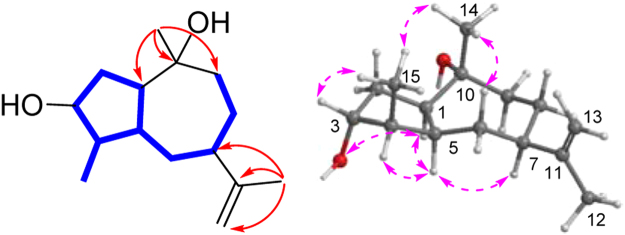
Selected COSY (), HMBC (), and NOESY () correlations of **1**.

Compound **1** was isolated as a colorless oil. The molecular formula C_15_H_26_O_2_ was established on the basis of the [M + H]^+^ ionic peak at *m/z* 239.2044 in its positive HRESIMS, requiring three indices of hydrogen deficiency. The ^1^H NMR spectroscopic data (Table 1) showed two singlet (*δ*_H_ 1.40 and 1.76) and one doublet (*δ*_H_ 1.08) methyls, one exomethylene (*δ*_H_ 4.83, 4.72), and one oxygenated methine (*δ*_H_ 4.22). These signals were resonance in the ^13^C NMR data (Table 2) as three methyls (*δ*_C_ 14.4, 19.9, and 29.4), one exocyclic methylene (*δ*_C_ 107.8), one oxygenated methine (*δ*_C_ 77.4). Altogether, the ^1^H and ^13^C NMR exhibited 15 carbons attributing to three methyls, five methylenes, five methines, and two nonprotonated carbons (one *sp*^2^ at *δ*_C_ 152.7 and one oxygenated *sp*^3^ at *δ*_C_ 73.0). Since an olefinic bond accounted for one unsaturation degree, a bicyclic framework was required for **1**. In the COSY spectrum, one spin coupling system from H-1 through H_2_-2 to H-3/H-4/H-5/H_2_-6/H-7/H_2_-8/H_2_-9, from H-5 to H-1, and from H-4 to H_3_-15 constructed a long fragment. In the HMBC spectrum, correlations from 14-Me (*δ*_H_ 1.40) to C-1 (*δ*_C_ 53.0), C-9 (*δ*_C_ 37.8), and C-10 (*δ*_C_ 73.0) and from 12-Me to C-7, C-11 (*δ*_C_ 152.7), and C-13 (*δ*_C_ 107.8) established **1** as a guaiane sesquiterpene (Fig. 2).

**Table 1:** ^1^H NMR spectroscopic data of **1**–**9** recorded at 400 MHz (*δ* in ppm, *J* in Hz within the parenthesis).

no.	**1** ^a^	**2** ^b^	**3** ^a^	**4** ^a^	**5** ^b^	**6** ^a^	**7** ^a^	**8** ^a^	**9** ^b^
1	2.93, dt (8.3, 9.9)	2.17, m	2.61, m	2.71, m	2.07, dd (9.9, 7.6)	2.51, m	2.55, m	3.20, m	2.12, m
2	2.22, m	1.73, m; 1.58, m	2.67, m; 2.26, m	3.18, ddd (19.7, 4.2, 1.3); 2.51, dd (19.4, 9.7)	4.27, q (7.2)	4.70, q (7.3)	4.64, q (6.9)	2.10, m; 1.76, m	1.72, m; 1.54, m
3	4.22, dt (9.5, 3.4)	1.73, m; 1.28, m	3.87, dt (6.8, 9.1)		1.70, m	1.96, m; 1.91, m	1.95, m	2.04, m; 1.92, td (12.8, 3.7)	1.72, m; 1.25, m
4	2.29, m	2.03, m	1.67, m	2.04, m	2.21, m	2.15, m	2.28, m		2.02, m
5	2.66, m	2.04, m	1.69, m	2.04, m	2.38, m	2.51, m	2.53, m	2.54, m	1.96, m
6	1.63, m; 1.47, td (13.3, 2.8)	1.40, m; 1.27, m	1.84, br d (12.9); 1.57, br t (11.5)	1.99, m; 1.48, m	1.42, m; 1.38, m	1.64, m	2.22, m; 1.29, m	2.63, dd (13.5, 3.2); 1.36, m	1.44, m; 1.08, m
7	2.59, td (10.3, 4.9)	2.34, m	2.10, br t (11.0)	2.29, td (10.9, 2.2)	2.19, m	2.39, m	2.27, m	2.51, m	2.05, m
8	2.09, m; 1.57, m	1.87, m; 1.43, m	1.76, m; 1.64, m	1.87, m; 1.54, m	1.75, m; 1.47, m	1.98, m; 1.68, m	2.16, m; 1.43, m	2.28, m; 1.49, m	1.87, m; 1.35, m
9	2.11, m; 1.80, m	1.91, m; 1.55, m	2.24, m; 1.98, td (13.5, 3.2)	2.19, m; 1.91, m	1.85, m; 1.69, m	2.15, m; 1.95, m	2.17, m; 1.91, m	2.21, m; 1.83, m	1.90, m; 1.51, m
11									2.30, m
12	1.76, s	1.70, s	1.76, s	1.75, s	1.70, s	4.47, t (1.4)	4.42, s	3.99, d (10.5); 3.89, d (10.5)	
13	4.83, d (1.7); 4.72, m	4.66, m; 4.58, m	4.84, d (1.5); 4.75, m	4.83, m; 4.74, m	4.65, m; 4.59, m	5.47, q (1.8); 5.08, m	1.36, s	1.41, s	1.10, d (7.0)
14	1.40, s	1.18, s	1.50, s	1.30, s	1.33, s	1.61, s	1.68, s	1.46, s	1.15, s
15	1.08, d (7.2)	0.92, d (6.8)	1.20, d (6.0)	1.08, d (6.4)	0.89, d (7.2)	0.83, d (7.3)	0.93, d (7.3)	1.54, s	0.92, d (7.0)
Ac							1.99, s		
2-OH							5.75, br s		
10-OH				5.78, br s			5.63, br s		
11-OH							5.96, br s		

^a^Measured in pyridine-*d*_5_. ^b^Measured in CD_3_OD.

**Table 2:** ^13^C NMR spectroscopic data of compounds **1**–**9**.

no.	**1** ^a^	**2** ^b^	**3** ^a^	**4** ^a^	**5** ^b^	**6** ^a^	**7** ^a^	**8** ^a^	**9** ^b^
1	53.0, CH	56.0, CH	49.6, CH	47.3, CH	62.7, CH	61.2, CH	62.6, CH	52.7, CH	56.1, CH
2	36.8, CH_2_	27.1, CH_2_	37.4, CH_2_	40.2, CH_2_	75.2, CH	74.1, CH	74.0, CH	25.9, CH_2_	27.0, CH_2_
3	77.4, CH	32.1, CH_2_	77.7, CH	220.6, C	43.1, CH_2_	42.5, CH_2_	43.0, CH_2_	39.6, CH_2_	31.8, CH_2_
4	48.0, CH	40.1, CH	49.0, CH	49.7, CH	37.0, CH	35.8, CH	36.3, CH	80.9, C	40.3, CH
5	44.9, CH	47.0, CH	46.0, CH	45.5, CH	46.2, CH	44.4, CH	46.0, CH	54.5, CH	47.3, CH
6	30.4, CH_2_	29.6, CH_2_	37.0, CH_2_	38.3, CH_2_	33.0, CH_2_	34.0, CH_2_	26.2, CH_2_	26.0, CH_2_	26.1, CH_2_
7	46.7, CH	47.1, CH	47.9, CH	44.9, CH	48.6, CH	43.7, CH	46.4, CH	45.5, CH	41.4, CH
8	29.7, CH_2_	29.7, CH_2_	32.0, CH_2_	32.5, CH_2_	31.1, CH_2_	31.6, CH_2_	26.2, CH_2_	25.9, CH_2_	28.0, CH_2_
9	37.8, CH_2_	36.8, CH_2_	46.2, CH_2_	45.0, CH_2_	42.0, CH_2_	43.8, CH_2_	40.7, CH_2_	38.0, CH_2_	35.4, CH_2_
10	73.0, C	75.2, C	73.9, C	73.3, C	75.7, C	74.3, C	73.9, C	73.4, C	75.3, C
11	152.7, C	153.4, C	152.5, C	152.1, C	153.5, C	157.9, C	73.4, C	75.3, C	47.0, CH
12	19.9, CH_3_	20.3, CH_3_	20.1, CH_3_	19.9, CH_3_	20.3, CH_3_	64.1, CH_2_	70.6, CH_2_	68.8, CH_2_	180.2, C
13	107.8, CH_2_	108.4, CH_2_	107.8, CH_2_	108.0, CH_2_	108.4, CH_2_	105.8, CH_2_	20.3, CH_3_	19.7, CH_3_	13.5, CH_3_
14	29.4, CH_3_	30.1, CH_3_	24.1, CH_3_	24.4, CH_3_	28.0, CH_3_	26.9, CH_3_	29.1, CH_3_	29.6, CH_3_	30.5, CH_3_
15	14.4, CH_3_	16.8, CH_3_	16.4, CH_3_	13.9, CH_3_	16.8, CH_3_	16.7, CH_3_	16.6, CH_3_	25.0, CH_3_	16.6, CH_3_
Ac							20.6, CH_3_ 170.7, C		

^a^Measured in pyridine-*d*_5_ at 100 MHz. ^b^Measured in CD_3_OD at 100 MHz.

**Figure 3:**
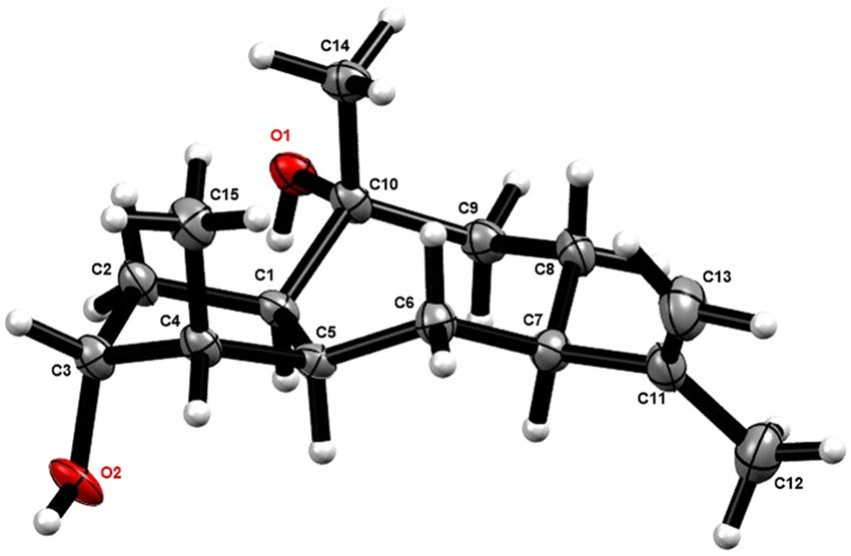
X-ray crystallographic structure of **1**.

In the NOESY spectrum, correlations from H-5 (*δ*_H_ 2.66) to H-1/H-4/H-7 (*δ*_H_ 2.59) and from Me-15 to Me-14 and H-3 disclosed that H-1, H-4, H-5, and H-7 were on the same face, whereas H-3, 14-Me, and 15-Me were on the opposite side (Fig. 2). This was unambiguously confirmed by the Cu-Kα X-ray crystallography (Fig. 3). Therefore, **1** was determined to be (1*R*,3*R*,4*R*,5*S*,7*R*,10*R*)-11(13)-en-3,10-dihydroxyguaiene, and named graphostromane A.

**Figure 4:**
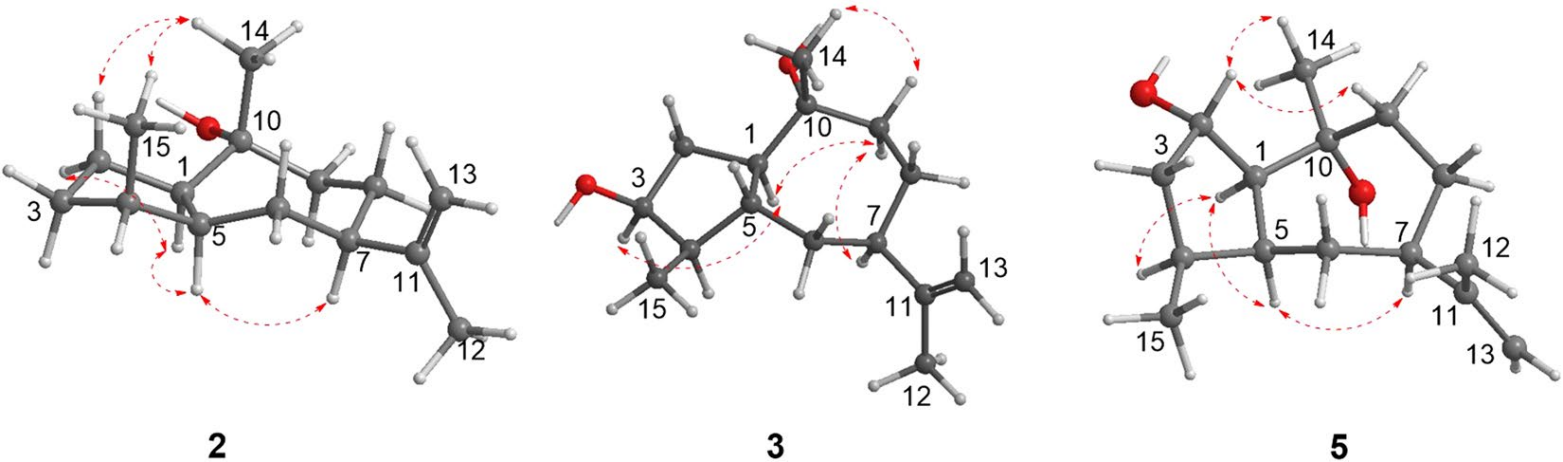
Key NOESY correlations of **2**, **3**, and **5**.

Compound **2** showed the formula molecular of C_15_H_26_O as established by the positive HRESIMS at *m/z* 223.2057 [M + H]^+^. The ^1^H and ^13^C NMR spectra were nearly identical to those of **1** except that a methylene (*δ*_C_ 32.1) instead of an oxygenated methine was located at C-3 position. This observation was evidenced by the HMBC correlation of 15-Me (*δ*_H_ 0.92) to the methylene unit. On the basis of its key NOESY correlations (Fig. 4) and the similar optical rotation value (${[\alpha ]}_{{\rm{D}}}^{25}$ −28.5 for **2**, while −22.0 for **1**), **2** was therefore deduced to be (1*R*,4*S*,5*S*,7*R*,10*R*)-11(13)-en-10-hydroxyguaiene, and named graphostromane B.

**Figure 5:**
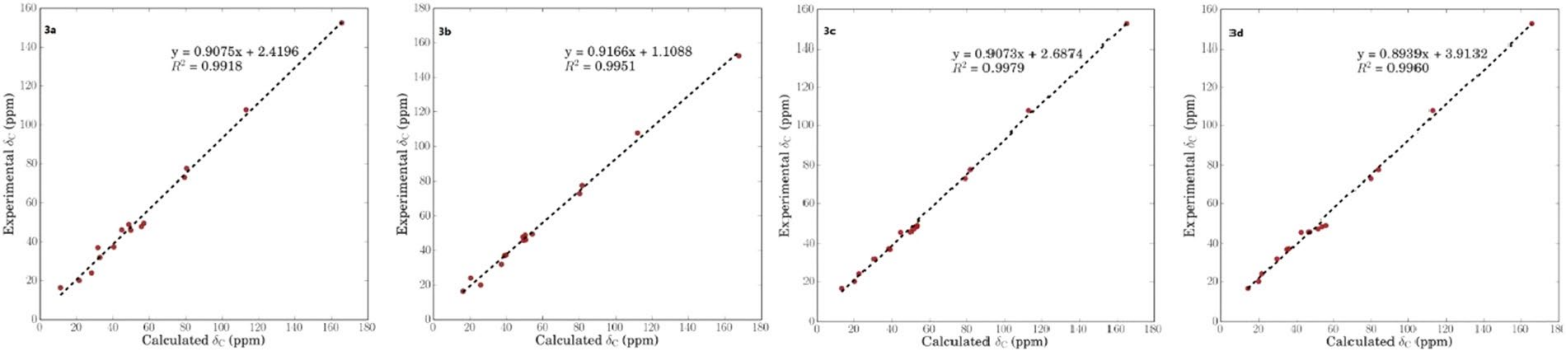
Calculated ^13^C NMR spectroscopic data of four possible stereoisomers of **3** (**3a**, **3b**, **3c**, and **3d**) at mPW1PW91/6-311 + G(2d,p) level in pyridine-*d*_*5*_.

**Figure 6:**
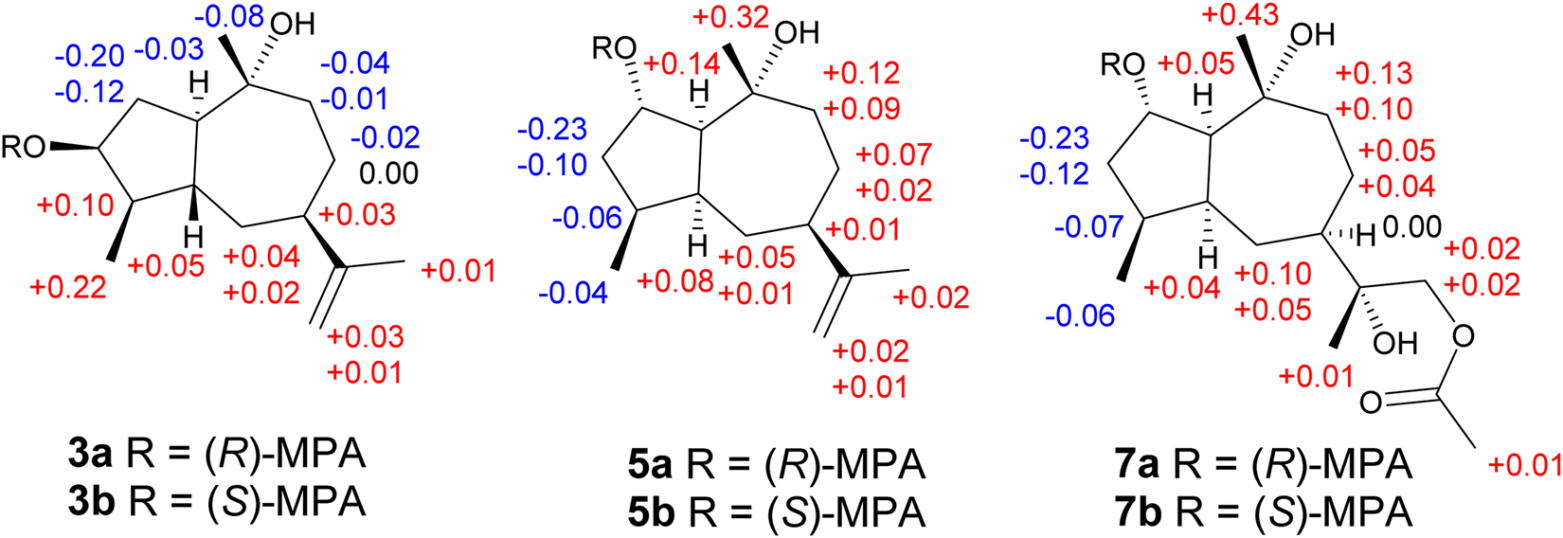
Δ*δ*_H_ (*δ*_H_^*R*^ ‒ *δ*_H_^*S*^) values of (*R*)- and (*S*)-MPA esters of **3**, **5**, and **7** in CDCl_3_.

Compound **3** showed the same molecule formula as that of  **1** by the positive HRESIMS at *m/z* 261.1829 (calcd for C_15_H_26_O_2_Na, 261.1830). Interestingly, it also exhibited almost the same ^1^H and ^13^C NMR spectra, except for the shielded chemical shift of H-3 (*δ*_H_ 3.87) and its peak pattern, indicating **3** and **1** might be C-3 stereoisomers. The NOESY correlations between H-3/H-1 (*δ*_H_ 2.61), H-1/H-9a (*δ*_H_ 1.98), H-9a/H-7, and H-9b (*δ*_H_ 2.24) to H_3_-14 (*δ*_H_ 1.50) deduced H-1, H-3, and H-7 were in the *α* orientations, while H_3_-14 was *β*-oriented. However, it is difficult to establish the relative configurations of C-4 and C-5 positions by the NOESY correlations because of the overlap signals of H-4 (*δ*_H_ 1.67) and H-5 (*δ*_H_ 1.69). Therefore, all four possible stereoisomers including a pair of *cis*-fused C-4 epimers [(1R*,3S*,4R*,5S*,7R*,10R*)-**3a** and (1R*,3S*,4S*,5S*,7R*,10R*)-**3b**] and a pair of *trans*-fused C-4 epimers [(1R*,3S*,4R*,5R*,7R*,10R*)-**3c** and (1R*,3S*,4S*,5R*,7R*,10R*)-**3d**] were subjected for the theoretical calculation of the CMR data at mPW1PW91/6-311 + G(2d,p) level using the IEFPCM model in pyridine-*d*_5_ by Gaussian 09. As shown in  Fig. 5, 3c displayed the smallest deviation, suggesting the relative configuration of **3** to be 1R*,3S*,4R*,5R*,7R*, and 10R*. By the modified Mosher’s method, C-3 was determined to be *S* configuration (Fig. 6)^21^. Based on the above evidences, **3** was then established to be (1*R*,3*S*,4*R*,5*R*,7*R*,10*R*)-11(13)-en-3,10-dihydroxyguaiene, and named graphostromane C.

Compound **4** was established the molecular formula C_15_H_24_O_2_ on the basis of its HRESIMS. The ^1^H and ^13^C NMR spectra were very similar to those of **3** except that a ketone group (*δ*_C_ 220.6) rather than a hydroxy unit was located at C-3. The assumption was corroborated by the HMBC relationships from 15-Me (*δ*_H_ 1.08) to the carbonyl carbon. Therefore, **4** was assigned as (1*R*,4*S*,5*S*,7*R*,10*R*)-3-oxo-11(13)-en-10-hydroxyguaiene, and named graphostromane D.

Compound **5** exhibited the same molecular formula as that of **1** according to its positive HRESIMS at *m/z* 261.1833 [M + Na]^+^. Close comparison of its ^1^H and ^13^C NMR spectra to those of **1** revealed a general similarity except that the hydroxy unit should be attached to C-2 instead of C-3 in **5**. This was confirmed by the HMBC correlations of 15-Me to the methylene unit at *δ*_C_ 43.1 of the C-3 position. In the NOESY spectrum, cross-peaks from H-9a (*δ*_H_ 1.85) to Me-14 (*δ*_H_ 1.33) and H-2 revealed H-2 was in *β* orientation (Fig. 4). By the modified Mosher’s method, C-2 was determined as *S*-configuration (Fig. 6). Accordingly, **5** was established to be (1*S*,2*S*,4*S*,5*S*,7*R*,10*R*)-11(13)-en-2,10-dihydroxyguaiene, and named graphostromane E.

Compound **6** was assigned the molecular formula C_15_H_26_O_3_ by the HRESIMS at *m/z* 277.1767 [M + Na]^+^. The ^1^H and ^13^C NMR spectra were nearly identical to those of **5**, except for an additional hydroxy unit at C-12 (*δ*_C_ 64.1). The assumption was evidenced by the HMBC correlations from H_2_-13 (*δ*_H_ 5.47, 5.08) to C-7 (*δ*_C_ 43.7), C-11 (*δ*_C_ 157.9), and C-12. Accordingly, **6** was determined to be (1*S*,2*S*,4*S*,5*S*,7*R*,10*R*)-11(13)-en-2,10,12-trihydroxyguaiene, and named graphostromane F.

**Figure 7:**
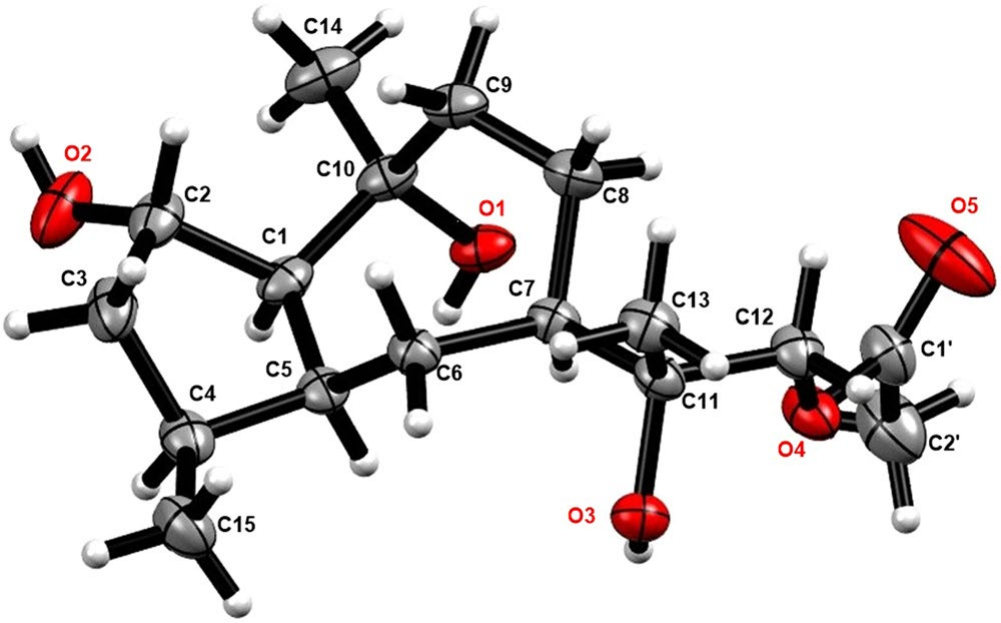
X-ray crystallographic structure of **7**.

Compound **7** gave a molecular formula C_17_H_30_O_5_ from its positive HRESIMS at *m/z* 337.1979 [M + Na]^+^. Its ^1^H and ^13^C NMR spectra were close to those of (1*S*,2*S*,4*S*,5*S*,7*R*,10*R*)-guaiane-2,10,11,12-tetraol (**10**)^22^, except for an additional acetyl group (*δ*_H_ 1.99; *δ*_C_ 20.6, 170.7) at C-12. This was evidenced by the HMBC cross-peaks from H_2_-12 (*δ*_H_ 4.42) to the carbonyl (*δ*_C_ 170.7) of the acetyl group. The absolute configuration of C-2 was established to be *S* on the basis of the modified Mosher’s method (Fig. 6), which was further corroborated by the X-ray single-crystal experiment (Fig. 7). Therefore, **7** was unambiguously determined to be (1*S*,2*S*,4*S*,5*S*,7*R*,10*R*,11*R*)-12-acetyl-2,10,11-trihydroxyguaiane, and named graphostromane G.

Compound **8** had the molecular formula of C_15_H_28_O_4_ as established by its HRESIMS. Analysis of the 1D and 2D NMR spectra established the structure of **8** was closely related to **10**, except that the hydroxy group was located at C-4 instead of C-2. This was evidenced by the HMBC correlations from 15-Me (*δ*_H_ 1.54) to C-3 (*δ*_C_ 39.6), C-4 (*δ*_C_ 80.9), and C-5 (*δ*_C_ 54.5). The NOE cross-peaks from H-2a (*δ*_H_ 2.10) to 14-Me (*δ*_H_ 1.46) and 15-Me revealed that 4-OH was in *α*-oriented. Therefore, **8** was identified to be (1*R*,4*R*,5*R*,7*R*,10*R*)-4,10,11-trihydroxyguaiane, and named graphostromane H.

**Figure 8:**
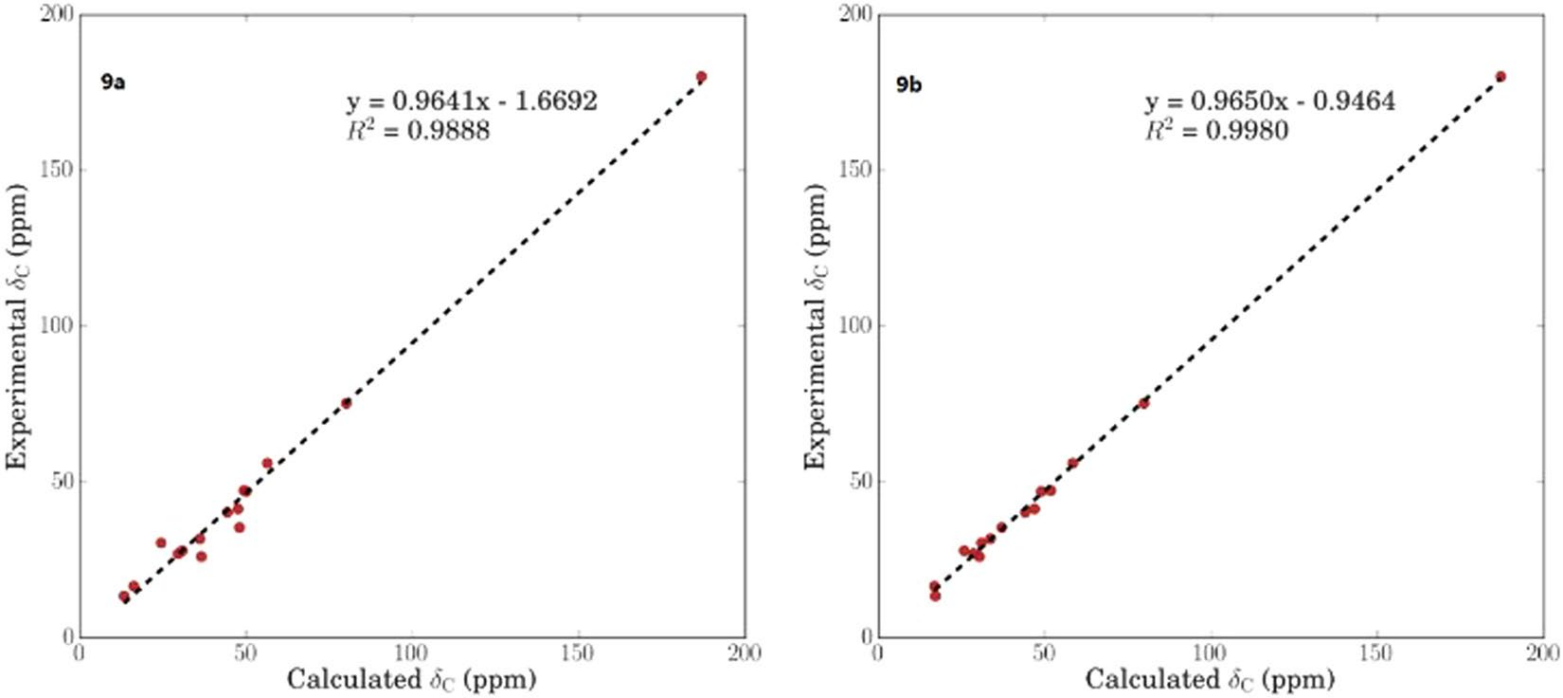
Calculated ^13^C NMR spectroscopic data of a pair of C-11 epimers of (1*R*,4*S*,5*S*,7*R*,10*R*,11*S*)-**9a** and (1*R*,4*S*,5*S*,7*R*,10*R*,11*R*)-**9b** at mPW1PW91/6-311 + G(2d,p) level in CD_3_OD.

Compound **9** was assigned the molecular formula C_15_H_26_O_3_ on the basis of the HRESIMS at *m/z* 277.1783 [M + Na]^+^. Its ^1^H and ^13^C NMR spectra were very similar to those of (1*R*,4*S*,5*S*,7*R*,10*R*,11*S*)-guaiane-10,11,12-triol (**11**)^15^ except that a hydroxy group was absent at C-11, while a carboxyl moiety (*δ*_C_ 180.2) instead of an oxymethylene was attached to C–11. This was corroborated by the HMBC correlations form 13-Me (*δ*_H_ 1.10) to C-7 (*δ*_C_ 41.4), C-11 (*δ*_C_ 47.0), and C–12 (*δ*_C_ 180.2). By the theoretical calculation of CMR spectrum, C-11 was assigned as *R*-configuration (Fig. 8). Therefore, **9** was established to be (1*R*,4*S*,5*S*,7*R*,10*R*,11*R*)-10-hydroxyguai-12-oic acid, and named graphostromane I.

By comparison of the NMR, MS, and OR data with those published in the literature, four known guaianes were identified as (1*S*,2*S*,4*S*,5*S*,7*R*,10*R*)-guaiane-2,10,11,12-tetraol (**10**)^22^, (1*R*,4*S*,5*S*,7*R*,10*R*,11*S*)-guaiane-10,11,12-triol (**11**)^15^, (1*R*,3*S*,4*R*,5*S*,7*R*,10*R*,11*S*)-guaiane-3,10,11,12-tetraol (**12**)^15^, and (1*R*,4*S*,5*S*,7*S*,9*R*,10*S*,11*R*)-guaiane-9,10,11,12-tetraol (**13**)^15^.

All isolates were evaluated for their anti-inflammatory activities against LPS-induced NO production in RAW264.7 macrophages (Table 3). Compound **6** exhibited remarkable anti-inflammatory activity with an IC_50_ value of 14.2 *μ*M, which was stronger than that of the positive control, aminoguanidine, with an IC_50_ of 23.4 *μ*M. In addition, **4**, **9**, and **13** showed weak anti-inflammatory activities with IC_50_ values of 72.9, 79.1, and 88.2 *μ*M, respectively.

**Table 3:** Anti-inflammatory activities of compounds **1**–**13** against LPS-stimulated NO production by in RAW 264.7 macrophages.

Compounds	IC_50_ (*μ*M)	CC_50_ (*μ*M)
**1**	310.1	>50
**2**	103.2	>50
**3**	112.6	>50
**4**	72.9	>50
**5**	138.2	>50
**6**	14.2	>50
**7**	101.0	>50
**8**	165.8	>50
**9**	79.1	>50
**10**	150.4	>50
**11**	141.0	>50
**12**	122.4	>50
**13**	88.2	>50
Aminoguanidine^a^	23.4	>50

^a^Positive control.

In conclusion, chemical investigation on the deep-sea-derived fungus *Graphostroma* sp. MCCC 3A00421 led to the isolation of 9 new (graphostromanes A–I, **1**–**9**) and 4 known (**10**–**13**) guaianes. They are first examples of guaiane sesquiterpenoids reported from the marine-derived fungi. Additionally, **6** showed potent anti-inflammatory activity against LPS-induced NO production in RAW264.7 macrophages, indicating its potential usage for anti-inflammatory drugs.

## Materials and Methods

### General Experimental Procedures

An automatic polarimeter Rudolph IV Autopol was used for recording optical rotation data at 25 °C. A Xevo G2 Q-TOF mass spectrometer was used for measuring HRESIMS. A Bruker Avance 400 MHz NMR spectrometer was used for measuring ^1^H, ^13^C, HSQC, COSY, HMBC, and NOESY spectra. Chemical shifts (*δ*) were expressed in ppm referring to the solvent peaks, and coupling constants are in Hz. A Bruker D8 Advance X-ray single-crystal diffractometer was used for measuring X-ray data with Cu Kα radiation. Column chromatography (CC) were performed on Sephadex LH-20 (18–110 *μ*m, Pharmacia, Uppsala, Sweden), silica gel (100–200 or 200–300 mush, Qingdao Marine Chemistry Co. Ltd, Qingdao, China), and ODS (50 *μ*m, Daiso, Japan). TLC precoated silica gel plates (GF254, Qingdao Marine Chemistry Co. Ltd, Qingdao, China) were used for TLC detection. All chemical reagents used were analytical grade.

### Fungal Identification and Fermentation

The fungus *Graphostroma* sp. MCCC 3A00421 was isolated from a hydrothermal sulfide deposit in August 2012 from the Atlantic Ocean (W 13.36°, S 15.17°) at a depth of −2721 m. It was identified to be *Graphostroma* genus on the basis of comparison of its ITS1-5.8S-ITS2 rRNA gene sequence (KM190888) with those deposited in GenBank of the NCBI using a BLAST searching tool. The voucher strain was deposited at the Marine Culture Collection of China (MCCC) with the accession number MCCC 3A00421.

The working strain was cultured on a PDA plate medium under 25 °C for 3 days. Then the fresh mycelia were inoculated into 30 Erlenmeyer flasks (1 L), each containing 120 mL distilled water and 80 g rice, and then statically fermented for 28 days at 25 °C.

### Extraction, Isolation, and Purification

After 28 days, the fermented cultures were extracted with EtOAc for three times. The EtOAc solution was evaporated under reduced pressure to get an organic extract, which was then partitioned between MeOH and petroleum ether (PE). The MeOH fraction was evaporated to get the defatted extract (7.0 g), which was separated by column chromatography (CC) over ODS eluting with gradient MeOH-H_2_O (5→100%) to yield 24 fractions (F1–F24). Fraction F7 (624 mg) was subjected to CC over Sephadex LH-20 (MeOH), followed by repeated CC on silica gel (CHCl_3_-MeOH, 15:1; EtOAc-MeOH, 50:1) to yield **10** (48.4 mg) and **12** (10.2 mg). Fraction F9 (420 mg) was purified by CC over Sephadex LH-20 (MeOH) to get three subfractions (SF9-1-9-3). SF9-1 was purified by CC over silica gel using PE-acetone (5:1), followed by Prep. TLC (CHCl_3_-MeOH, 8:1) to provide **7** (12.6 mg). SF9-3 was subjected to CC over silica gel (CHCl_3_-MeOH, 15:1) to afford **8** (8.2 mg). Compounds **6** (18.8 mg) and **13** (24.2 mg) were isolated from fraction F10 (603 mg) by CC over Sephadex LH-20 (MeOH) and silica gel (PE-acetone, 3:1; PE-EtOAc, 3:1→1:1). Fraction F14 (389 mg) was subjected to CC over silica gel using gradient PE-EtOAc to give seven subfractions (SF14-1‒SF14-7). SF14-2 was chromatographed on Sephadex LH-20 (MeOH) to yield **4** (26.0 mg). SF14-3 was purified by Prep. TLC (PE-acetone, 2:1) to get **1** (14.7 mg). Compound **3** (10.2 mg) was isolated from SF14-5 by Prep. TLC (EtOAc-acetone, 30:1). SF14-6 was separated by CC over Sephadex LH-20 (MeOH) and silica gel (CHCl_3_-MeOH, 20:1) to yield **11** (39.2 mg). Fraction F16 (462 mg) was subjected to CC on Sephadex LH-20 (MeOH) and silica gel (CHCl_3_-MeOH, 100:1→1:1; EtOAc-MeOH, 200:1→10:1; and PE-acetone, 5:1) to yield **2** (23.0 mg), **5** (15.5 mg), and **9** (11.6 mg).

*Graphostromane A (****1****)*: colorless oil; ${[\alpha ]}_{{\rm{D}}}^{25}$ −22.0 (*c* 0.58, MeOH); ^1^H and ^13^C NMR data, Tables 1 and 2; HRESIMS *m/z* 239.2044 [M + H]^+^ (calcd for C_15_H_27_O_2_, 239.2011).

*Graphostromane B (****2****)*: colorless oil; ${[\alpha ]}_{{\rm{D}}}^{25}$ −28.5 (*c* 2.16, MeOH); ^1^H and ^13^C NMR data, Tables 1 and 2; HRESIMS *m/z* 223.2057 [M + H]^+^ (calcd for C_15_H_27_O, 223.2062).

*Graphostromane C (****3****)*: colorless oil; ${[\alpha ]}_{{\rm{D}}}^{25}$ −8.5 (*c* 0.25, MeOH); ^1^H and ^13^C NMR data, Tables 1 and 2; HRESIMS *m/z* 261.1829 [M + Na]^+^ (calcd for C_15_H_26_O_2_Na, 261.1830).

*Graphostromane D (****4****)*: colorless oil; ${[\alpha ]}_{{\rm{D}}}^{25}$ +15.4 (*c* 0.73, MeOH); ^1^H and ^13^C NMR data, Tables 1 and 2; HRESIMS *m/z* 237.1851 [M + H]^+^ (calcd for C_15_H_25_O_2_, 237.1855).

*Graphostromane E (***5***)*: colorless oil; ${[\alpha ]}_{{\rm{D}}}^{25}$ +8.13 (*c* 0.39, MeOH); ^1^H and ^13^C NMR data, Tables 1 and 2; HRESIMS *m/z* 261.1833 [M + Na]^+^ (calcd for C_15_H_26_O_2_Na, 261.1830).

*Graphostromane F (****6****)*: colorless oil; ${[\alpha ]}_{{\rm{D}}}^{25}$ +3.41 (*c* 0.44, MeOH); ^1^H and ^13^C NMR data, Tables 1 and 2; HRESIMS *m/z* 277.1767 [M + Na]^+^ (calcd for C_15_H_26_O_3_Na, 277.1780).

*Graphostromane G (****7****)*: colorless oil; ${[\alpha ]}_{{\rm{D}}}^{25}$ +6.0 (*c* 0.56, MeOH); ^1^H and ^13^C NMR data, Tables 1 and 2; HRESIMS *m/z* 337.1979 [M + Na]^+^ (calcd for C_17_H_30_O_5_Na, 337.1991).

*Graphostromane H (****8****)*: colorless oil; ${[\alpha ]}_{{\rm{D}}}^{25}$ −21.0 (*c* 0.25, MeOH); ^1^H and ^13^C NMR data, Tables 1 and 2; HRESIMS *m/z* 295.1878 [M + Na]^+^ (calcd for C_15_H_28_O_4_Na, 295.1885).

*Graphostromane I (****9****)*: colorless oil; ${[\alpha ]}_{{\rm{D}}}^{25}$ −10.3 (*c* 0.12, MeOH); ^1^H and ^13^C NMR data, Tables 1 and 2; HRESIMS *m/z* 277.1783 [M + Na]^+^ (calcd for C_15_H_26_O_3_Na, 277.1780).

Compound **10**: ^13^C NMR (CD_3_OD, 100 MHz) *δ*_C_ 63.4 (CH-1), 75.1 (CH-2), 43.3 (CH_2_-3), 37.3 (CH-4), 47.3 (CH-5), 25.8 (CH_2_-6), 45.7 (CH-7), 26.4 (CH_2_-8), 39.5 (CH_2_-9), 75.4 (C-10), 76.6 (C-11), 69.2 (CH_2_-12), 19.0 (Me-13), 29.6 (Me-14), 16.7 (Me-15).

### Preparation of (*R*)- and (*S*)-MPA Esters of Compounds **3**,** 5**, and **7**

Compound **3** (2.0 mg) was dissolved in CHCl_3_ (600 *µ*L). Then (*R*)-MPA (2.5 mg), DCC (2.5 mg), and DMAP (2.5 mg) were added. After stirred 16 h at room temperature, the reactive products were subjected to CC over silica gel (PE-acetone, 3:1) to give the *R*-MPA ester **3a** (1.7 mg). Similarly, the *S*-MPA ester **3b** (1.9 mg) was obtained from (*S*)-MPA. Analogue treatment of compounds **5** and **7** separately with (*R*)-MPA and (*S*)-MPA obtained (*R*)-MPA esters (**5a** and **7a**) and (*S*)-MPA esters (**5b** and **7b**), respectively.

(*R*)-*MPA ester of* **3** (**3*a***): ^1^H NMR (CDCl_3_, 400 MHz) *δ*_H_ 7.32–7.47 (5 H, m, phenyl protons), 4.78 (1 H, s, CH of MPA), 3.43 (3 H, s, OMe of MPA), 2.25 (1 H, m, H-1), 1.44 (1 H, m, H-2a), 2.22 (1 H, m, H-2b), 4.68 (1 H, m, H-3), 1.61 (1 H, m, H-4), 1.66 (1 H, m, H-5), 1.36 (1 H, m, H-6a), 1.69 (1 H, m, H-6b), 1.96 (1 H, dt, *J* = 2.2, 11.5 Hz, H-7), 1.46 (1 H, m, H-8a), 1.71 (1 H, m, H-8b), 1.63 (1 H, m, H-9a), 1.85 (1 H, ddd, *J* = 2.6, 4.7, 8.1 Hz, H-9b), 1.70 (3 H, s, Me-12), 4.62 (1 H, br s, H-13a), 4.65 (1 H, br s, H-13b), 1.18 (3 H, s, Me-14), 0.91 (3 H, d, *J* = 6.2 Hz, Me-15).

(*S*)-*MPA ester of* **3** (**3*b***): ^1^H NMR (CDCl_3_, 400 MHz) *δ*_H_ 7.33–7.47 (5 H, m, phenyl protons), 4.77 (1 H, s, CH of MPA), 3.44 (3 H, s, OMe of MPA), 2.28 (1 H, m, H-1), 1.64 (1 H, m, H-2a), 2.34 (1 H, m, H-2b), 4.63 (1 H, m, H-3), 1.51 (1 H, m, H-4), 1.61 (1 H, m, H-5), 1.34 (1 H, m, H-6a), 1.65 (1 H, m, H-6b), 1.93 (1 H, t, *J* = 10.8 Hz, H-7), 1.48 (1 H, m, H-8a), 1.71 (1 H, m, H-8b), 1.64 (1 H, m, H-9a), 1.89 (1 H, m, H-9b), 1.69 (3 H, s, Me-12), 4.61 (1 H, br s, H-13a), 4.64 (1 H, br s, H-13b), 1.26 (3 H, s, Me-14), 0.69 (3 H, d, *J* = 6.5 Hz, Me-15).

(*R*)-*MPA ester of* **5** (**5*a***): ^1^H NMR (CDCl_3_, 400 MHz) *δ*_H_ 7.32–7.47 (5 H, m, phenyl protons), 4.78 (1 H, s, CH of MPA), 3.43 (3 H, s, OMe of MPA), 2.35 (1 H, m, H-1), 5.25 (1 H, m, H-2), 1.51 (1 H, m, H-3a), 1.64 (1 H, m, H-3b), 2.16 (1 H, m, H-4), 2.30 (1 H, m, H-5), 1.23 (1 H, m, H-6a), 1.45 (1 H, m, H-6b), 2.11 (1 H, m, H-7), 1.45 (1 H, m, H-8a), 1.75 (1 H, m, H-8b), 1.67 (1 H, m, H-9a), 1.82 (1 H, m, H-9b), 1.69 (3 H, s, Me-12), 4.61 (1 H, br s, H-13a), 4.65 (1 H, br s, H-13b), 1.22 (3 H, s, Me-14), 0.87 (3 H, d, *J* = 7.0 Hz, Me-15).

(*S*)-*MPA ester of* **5** (**5*b***): ^1^H NMR (CDCl_3_, 400 MHz) *δ*_H_ 7.31–7.44 (5 H, m, phenyl protons), 4.73 (1 H, s, CH of MPA), 3.41 (3 H, s, OMe of MPA), 2.21 (1 H, m, H-1), 5.15 (1 H, m, H-2), 1.74 (2 H, m, H-3), 2.22 (2 H, m, H-4 and H-5), 1.17 (1 H, m, H-6a), 1.44 (1 H, m, H-6b), 2.10 (1 H, dt, *J* = 4.1, 11.4 Hz, H-7), 1.38 (1 H, m, H-8a), 1.73 (2 H, m, H-8b and H-9b), 1.55 (1 H, t, *J* = 10.1 Hz, H-9a), 1.67 (3 H, s, Me-12), 4.60 (1 H, br s, H-13a), 4.63 (1 H, br s, H-13b), 0.90 (3 H, s, Me-14), 0.91 (3 H, d, *J* = 7.0 Hz, Me-15).

(*R*)-*MPA ester of* **7** (**7*a***): ^1^H NMR (CDCl_3_, 400 MHz) *δ*_H_ 7.32–7.44 (5 H, m, phenyl protons), 4.74 (1 H, s, CH of MPA), 3.41 (3 H, s, OMe of MPA), 2.31 (1 H, t, *J* = 6.9 Hz, H-1), 5.17 (1 H, dt, *J* = 2.9, 7.6 Hz, H-2), 1.45 (1 H, m, H-3a), 1.68 (1 H, m, H-3b), 2.16 (1 H, m, H-4), 2.18 (1 H, m, H-5), 0.91 (1 H, m, H-6a), 1.81 (1 H, m, H-6b), 1.77 (1 H, m, H-7), 1.19 (1 H, m, H-8a), 1.76 (1 H, m, H-8b), 1.59 (1 H, m, H-9a), 1.82 (1 H, m, H-9b), 1.06 (3 H, s, Me-12), 3.97 (1 H, d, *J* = 11.5 Hz, H-13a), 4.06 (1 H, d, *J* = 11.5 Hz, H-13b), 1.14 (3 H, s, Me-14), 0.90 (3 H, d, *J* = 6.5 Hz, Me-15).

(*S*)-*MPA ester of* **7** (**7*b***): ^1^H NMR (CDCl_3_, 400 MHz) *δ*_H_ 7.32–7.42 (5 H, m, phenyl protons), 4.71 (1 H, s, CH of MPA), 3.40 (3 H, s, OMe of MPA), 2.16 (1 H, m, H-1), 5.05 (1 H, dt, *J* = 2.8, 7.7 Hz, H-2), 1.68 (1 H, m, H-3a), 1.80 (1 H, m, H-3b), 2.23 (1 H, m, H-4), 2.21 (1 H, m, H-5), 0.86 (1 H, m, H-6a), 1.71 (2 H, m, H-6b and H-8b), 1.77 (1 H, m, H-7), 1.15 (1 H, m, H-8a), 1.46 (1 H, m, H-9a), 1.72 (1 H, m, H-9b), 1.05 (3 H, s, Me-12), 3.95 (1 H, d, *J* = 11.5 Hz, H-13a), 4.04 (1 H, d, *J* *=* 11.5 Hz, H-13b), 0.71 (3 H, s, Me-14), 0.96 (3 H, d, *J* = 6.8 Hz, Me-15).

### X-ray Crystal Data of Compounds **1** and** 7**

Graphostromane A (**1**) was obtained as colorless crystals. The monoclinic crystals (0.1 × 0.2 × 0.8 mm^3^) was recorded on a Bruker D8 Advance X-ray single-crystal diffractometer with Cu Kα radiation. Crystal data of **1**: empirical formula C_15_H_26_O_2_, M = 236.36; space group P2_1_, unit cell dimensions a = 6.3808 (4) Å, b = 7.7943 (4) Å, c = 13.9771 (8) Å, *α* = 90.00°, *β* = 101.329 (6)°, *γ* = 90.00°, V = 681.59 (7) Å^3^, Z = 2, D_calcd_ = 1.1516 g/cm^3^, *µ* = 0.579 mm^−1^, F (000) = 260.8; A total of 6871 reflections were collected in the range of 6.44° < 2θ < 124.16°, of which 2128 independent reflections [R_int_ = 0.0548, R_sigma_ = 0.0552] were used for the analysis. The structure was solved by the direct methods with the SHELXL-97 program and refined using full-matrix least-squares difference Fourier techniques. The final R indexes [all data] gave R_1_ = 0.0642, wR_2_ = 0.1712 and the Flack parameter = −0.1 (3). Crystallographic data of **1** have been deposited in the Cambridge Crystallographic Data Center (deposition number CCDC 1529237).

Colorless crystals of **7** were obtained from MeOH. The monoclinic crystals (0.3 × 0.4 × 0.8 mm^3^) was measured on a Bruker D8 Advance X-ray single-crystal diffractometer with Cu Kα radiation. Crystal data of **7**: empirical formula C_17_H_30_O_5_, M = 314.42; space group C2, unit cell dimensions a = 12.2072 (4) Å, b = 14.3161 (3) Å, c = 13.4457 (4) Å, *α* = *γ* = 90.00°, *β* = 114.561 (4)°, Volume = 2137.16 (12) Å^3^, Z = 9, D_calcd_ = 1.173 g/cm^3^, *µ* = 0.774 mm^−1^, F (000) = 828.0; A total of 11239 reflections were collected in the range of 7.228° < 2θ < 123.85°, of which 3337 independent reflections [R_int_ = 0.0330, R_sigma_ = 0.0280] were used for the analysis. The final R indexes [all data] gave R_1_ = 0.0368, wR_2_ = 0.1006 and the Flack parameter = 0.04 (7). Crystallographic data of **7** have been deposited in the Cambridge Crystallographic Data Center (deposition number CCDC 1577811).

### Theoretical Calculation of CMR Data

The calculated CMR data of **3** and **9** were carried out by Gaussian 09^23^. Conformational analyses were initially performed using Confab^24^ with MMFF94 force field for configurations of both compounds. The conformers, which were chosen for CMR calculations with Boltzmann-population over 1%, were firstly optimized at PM6 by semi-empirical theory method to filter some conformers with low Boltzmann-populations. Then, the remaining conformers were optimized at B3LYP/6-31 + G(d,p) in gas phase. The CMR calculation was conducted by the Gauge-Including Atomic Orbitals (GIAO) method at mPW1PW91/6-311 + G(2d,p) level using the IEFPCM model in pyridine for **3**, whereas in MeOH for **9**, respectively. Finally, the TMS-corrected ^13^C NMR chemical shift values were averaged according to Boltzmann distribution for each conformer and fitting to the experimental values by linear regression. The calculated CMR chemical shift values of TMS in pyridine and in MeOH were 187.3194 ppm and 187.3772 ppm, respectively.

### Anti-Inflammatory Assay

This experiment was conducted according the reported procedure^25^.

## Electronic supplementary material

Supplementary Information
