# Supplementary material for: New anti-inflammatory guaianes from the Atlantic hydrotherm-derived fungus Graphostroma sp. MCCC 3A00421
Source: Sci Rep. 2018 Jan 11;8:530. doi: 10.1038/s41598-017-18841-6 (PMC5765142; doi:10.1038/s41598-017-18841-6)
Supplement: Supplementary file 1 — Supplementary Information [file 41598_2017_18841_MOESM1_ESM.pdf]

Supporting Information

New anti-inflammatory guaianes from the Atlantic hydrotherm-derived fungus *Graphostroma* sp. MCCC 3A00421

Siwen Niu, Chun-Lan Xie, Jin-Mei Xia, Zhu-Hua Luo, Zongze Shao & Xian-Wen Yang*

State Key Laboratory Breeding Base of Marine Genetic Resources, Key Laboratory of Marine Genetic Resources, Fujian Key Laboratory of Marine Genetic Resources, South China Sea Bio-Resource Exploitation and Utilization Collaborative Innovation Center, Third Institute of Oceanography, State Oceanic Administration, 184 Daxue Road, Xiamen 361005, People's Republic of China

Contents

Figure S1-1. ^{1}H NMR spectrum of **1** in pyridine-d_5 (400 MHz).

Figure S1-2. ^{13}C NMR spectrum of **1** in pyridine-d_5 (100 MHz).

Figure S1-3. HSQC NMR spectrum of **1** in pyridine-d_5.

Figure S1-4. COSY Spectrum of **1** in pyridine-d_5.

Figure S1-5. HMBC NMR spectrum of **1** in pyridine-d_5.

Figure S1-6. NOESY spectrum of **1** in pyridine-d_5.

Figure S2-1. ^{1}H NMR spectrum of **2** in CD_3OD (400 MHz).

Figure S2-2.^{13}C NMR spectrum of **2** in CD_3OD (100 MHz).

Figure S3-1. ^{1}H NMR spectrum of **3** in pyridine-d_5 (400 MHz).

Figure S3-2. ^{13}C NMR spectrum of **3** in pyridine-d_5 (100 MHz).

Figure S3-3. ^{1}H NMR spectrum of *R*-MPA ester of **3** (**3a**) in $CDCl_3$ (400 MHz).

Figure S3-4. ^{1}H NMR spectrum of *S*-MPA ester of **3** (**3b**) in $CDCl_3$ (400 MHz).

Figure S4-1. ^{1}H NMR spectrum of **4** in pyridine-d_5 (400 MHz).

Figure S4-2. ^{13}C NMR spectrum of **4** in pyridine-d_5 (100 MHz).

Figure S5-1. ^{1}H NMR spectrum of **5** in CD_3OD (400 MHz).

Figure S5-2. ^{13}C NMR spectrum of **5** in CD_3OD (100 MHz).

Figure S5-3. ^{1}H NMR spectrum of *R*-MPA ester of **5** (**5a**) in $CDCl_3$ (400 MHz).

Figure S5-4. ^{1}H NMR spectrum of *S*-MPA ester of **5** (**5b**) in $CDCl_3$ (400 MHz).

Figure S5-5. NOESY spectrum of **5** in CD_3OD (400 MHz).

Figure S6-1. ^{1}H NMR spectrum of **6** in pyridine-d_5 (400 MHz).

Figure S6-2. ^{13}C NMR spectrum of **6** in pyridine-d_5 (100 MHz).

Figure S7-1. ^{1}H NMR spectrum of **7** in pyridine-d_5 (400 MHz).

Figure S7-2. ^{13}C NMR spectrum of **7** in pyridine-d_5 (100 MHz).

Figure S7-3. ^{1}H NMR spectrum of *R*-MPA ester of **7** (**7a**) in $CDCl_3$ (400 MHz).

Figure S7-4. ^{1}H NMR spectrum of *S*-MPA ester of **7** (**7b**) in $CDCl_3$ (400 MHz).

Figure S7-5. NOESY spectrum of **7** in pyridine-d_5 (400 MHz).

Figure S8-1. ^{1}H NMR spectrum of **8** in pyridine-d_5 (400 MHz).

Figure S8-2. ^{13}C NMR spectrum of **8** in pyridine-d_5 (100 MHz).

Figure S9-1. ^{1}H NMR spectrum of **9** in CD_3OD (400 MHz).

Figure S9-2. ^{13}C NMR spectrum of **9** in CD_3OD (100 MHz).

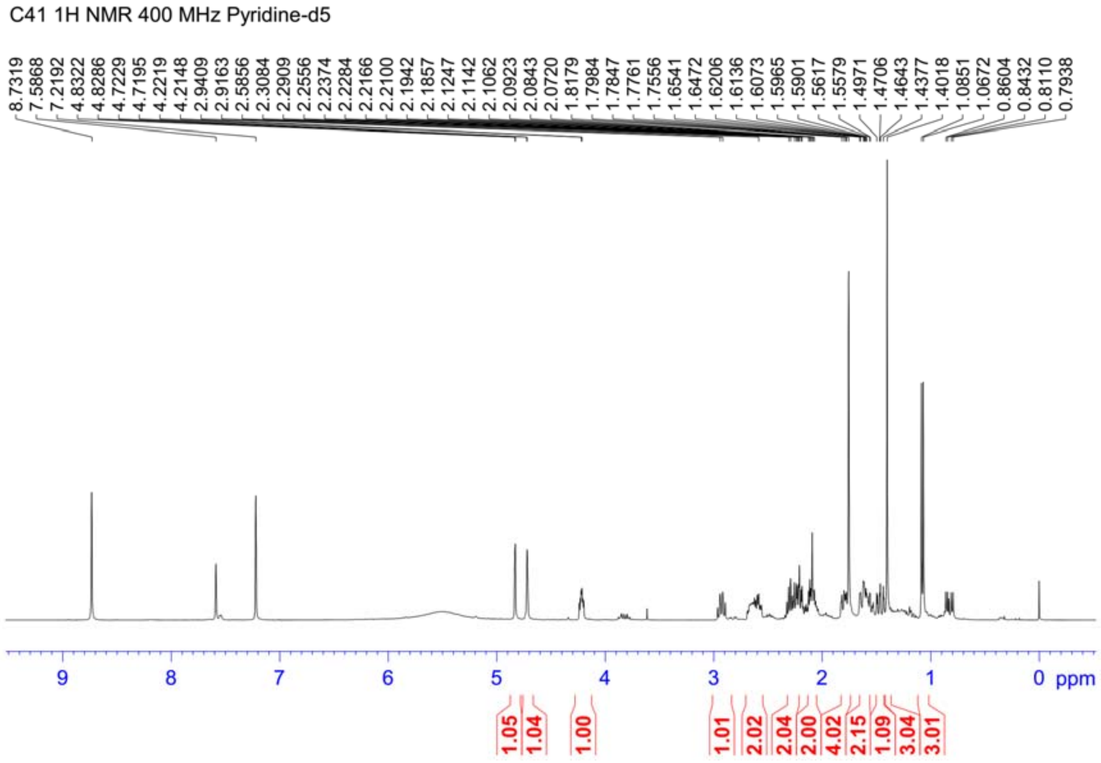

Figure S1-1. ^{1}H NMR spectrum of **1** in pyridine-d_5 (400 MHz).

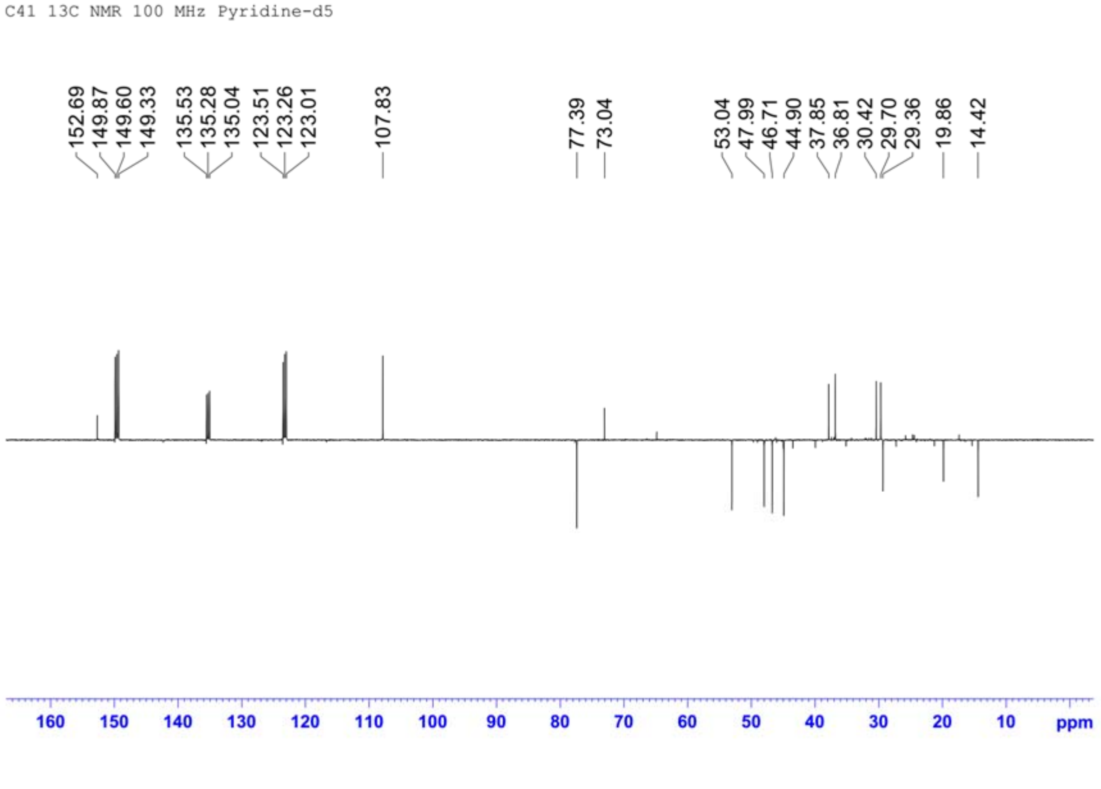

Figure S1-2. ^{13}C NMR spectrum of **1** in pyridine-d_5 (100 MHz).

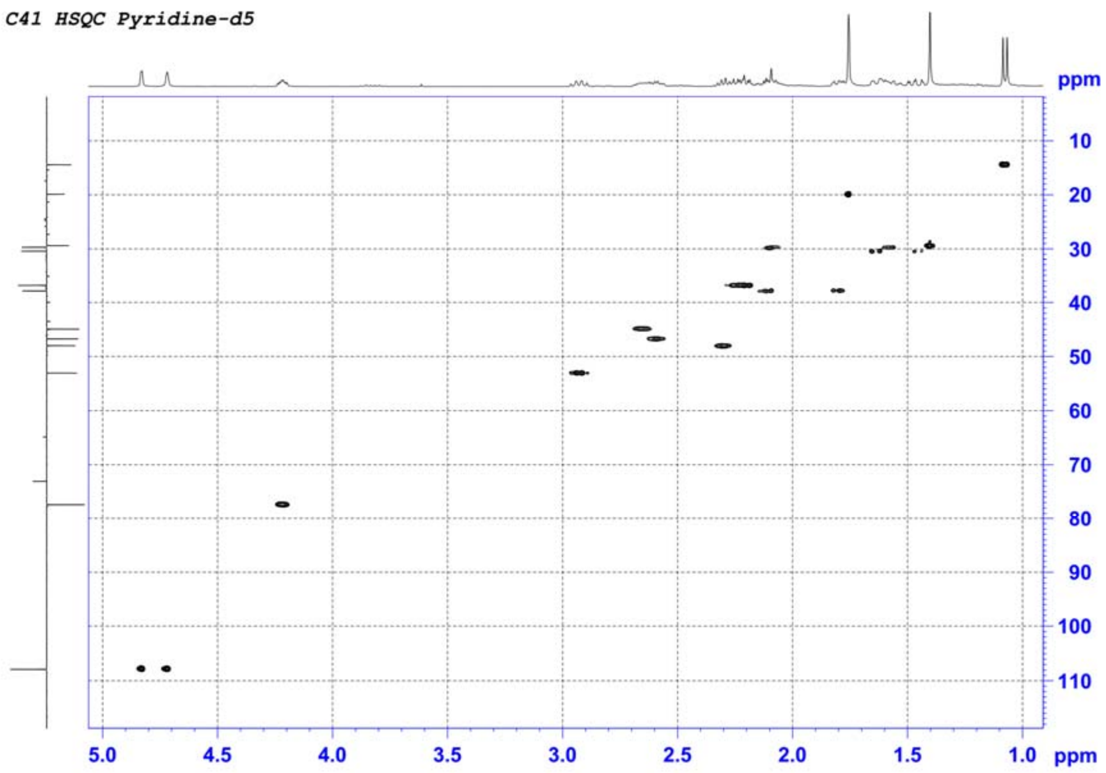

Figure S1-3. HSQC NMR spectrum of **1** in pyridine-d_5.

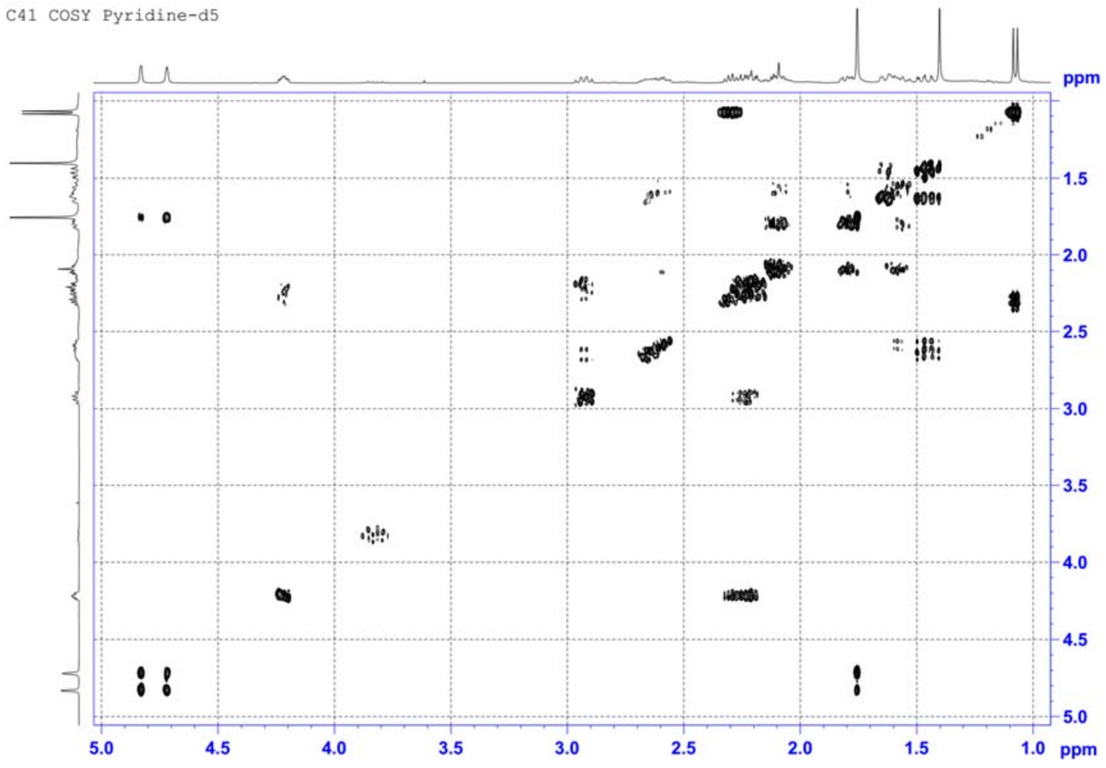

Figure S1-4. COSY Spectrum of **1** in pyridine-d_5.

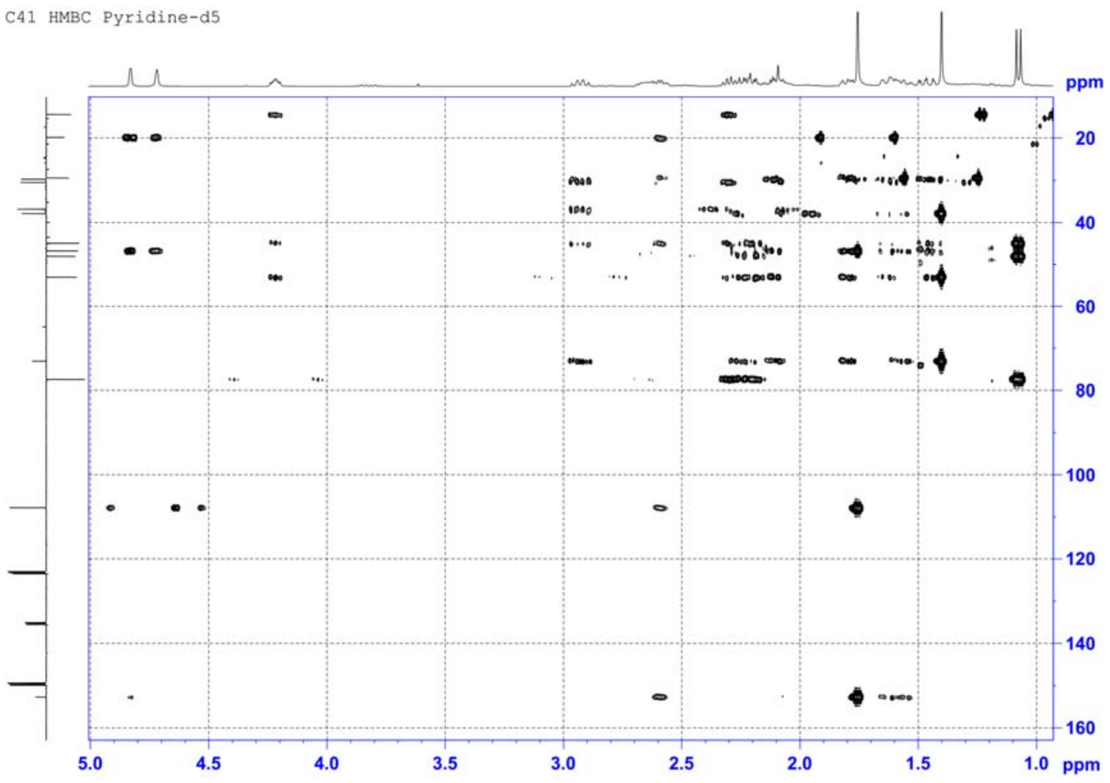

Figure S1-5. HMBC NMR spectrum of **1** in pyridine-d_5.

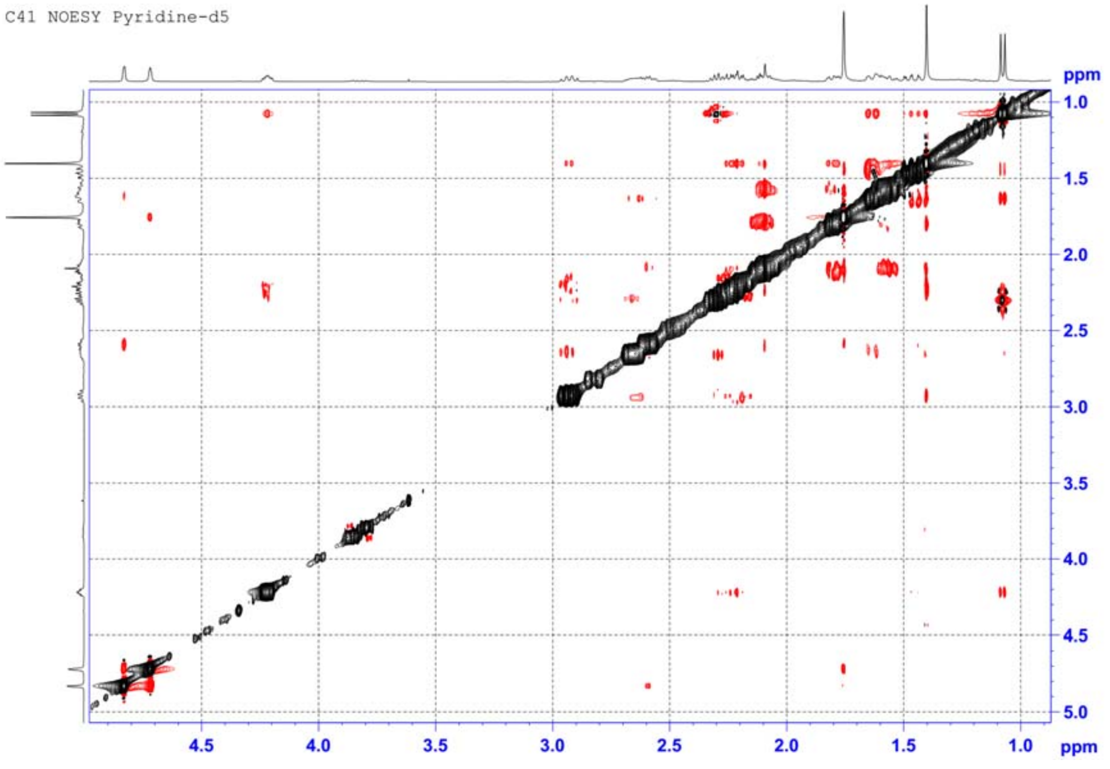

Figure S1-6. NOESY Spectrum of **1** in pyridine-d_5.

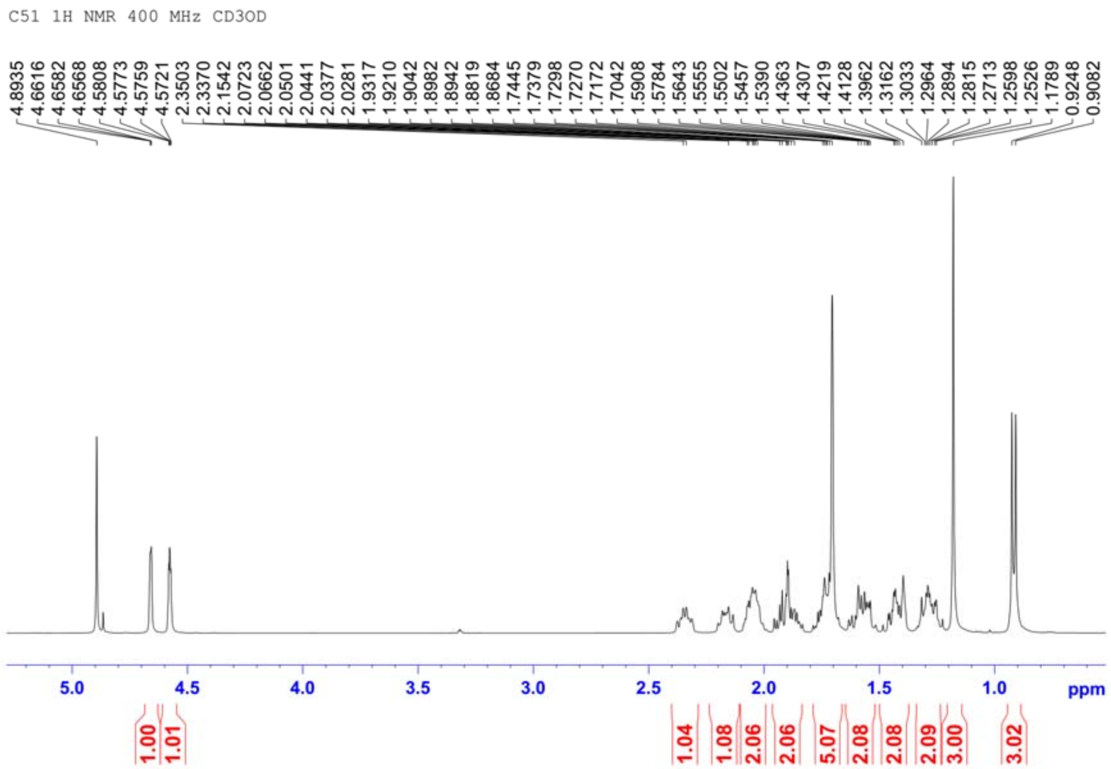

Figure S2-1. ^{1}H NMR spectrum of **2** in CD_3OD (400 MHz).

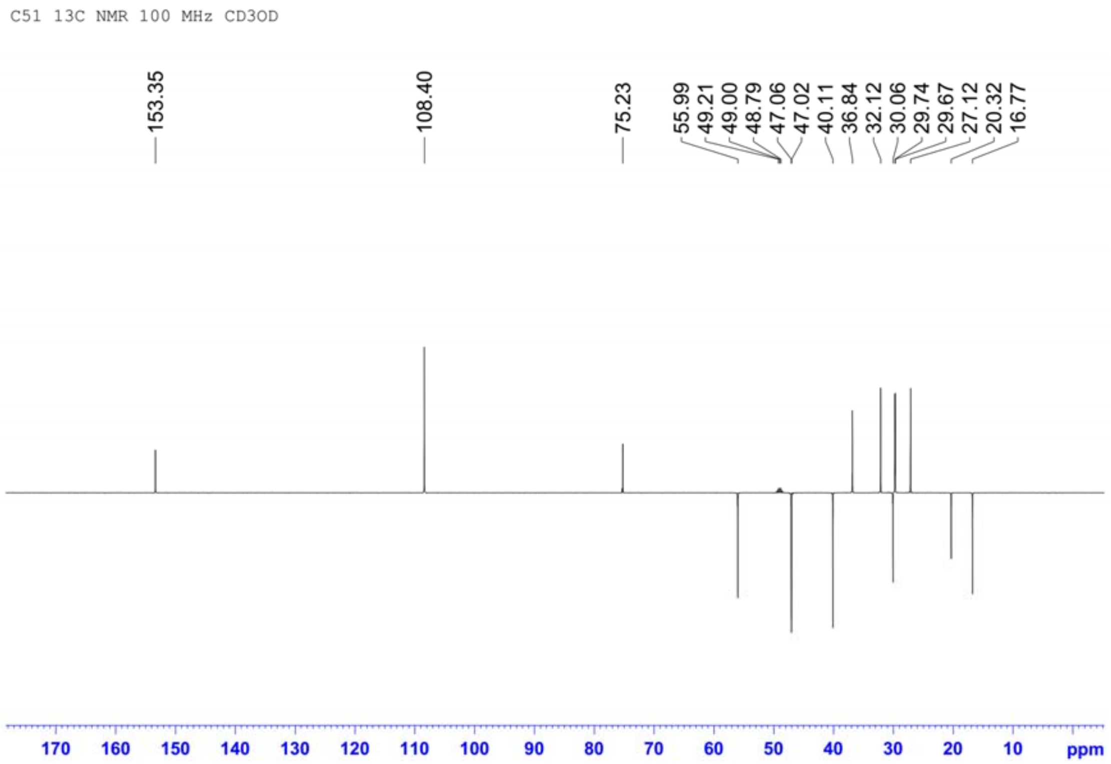

Figure S2-2. ^{13}C NMR spectrum of **2** in CD_3OD (100 MHz).

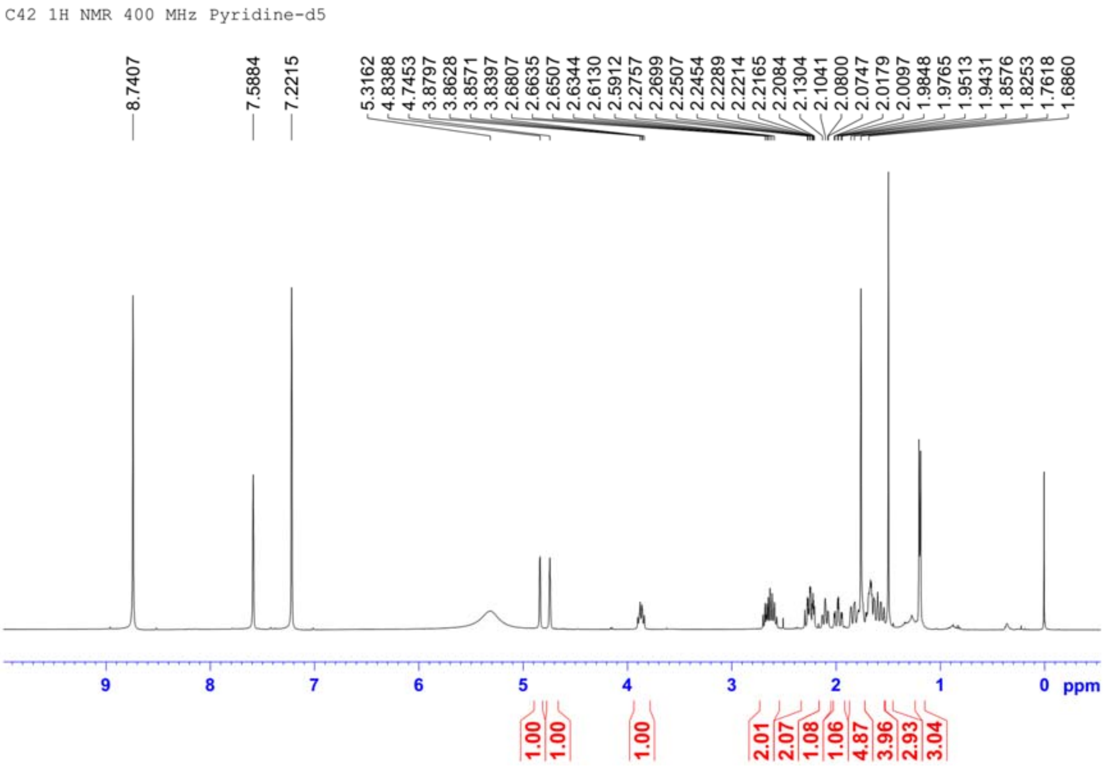

Figure S3-1. ^{1}H NMR spectrum of **3** in pyridine-*d*$_5$ (400 MHz).

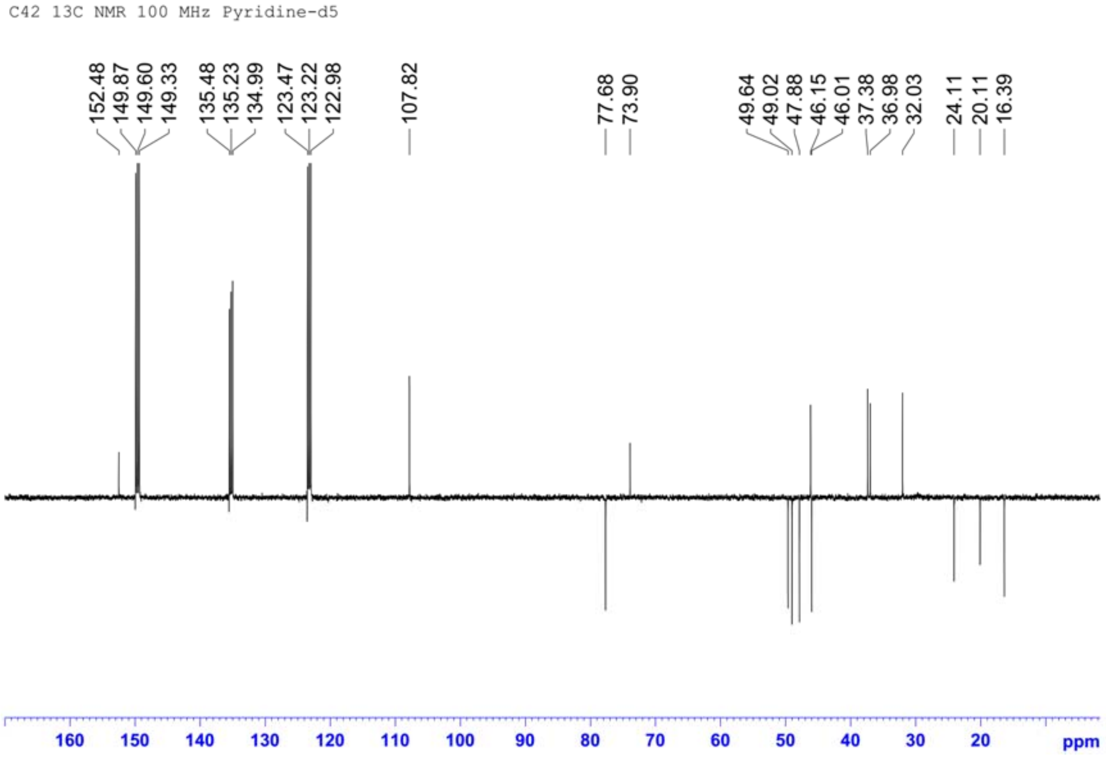

Figure S3-2. ^{13}C NMR spectrum of **3** in pyridine-*d*$_5$ (100 MHz).

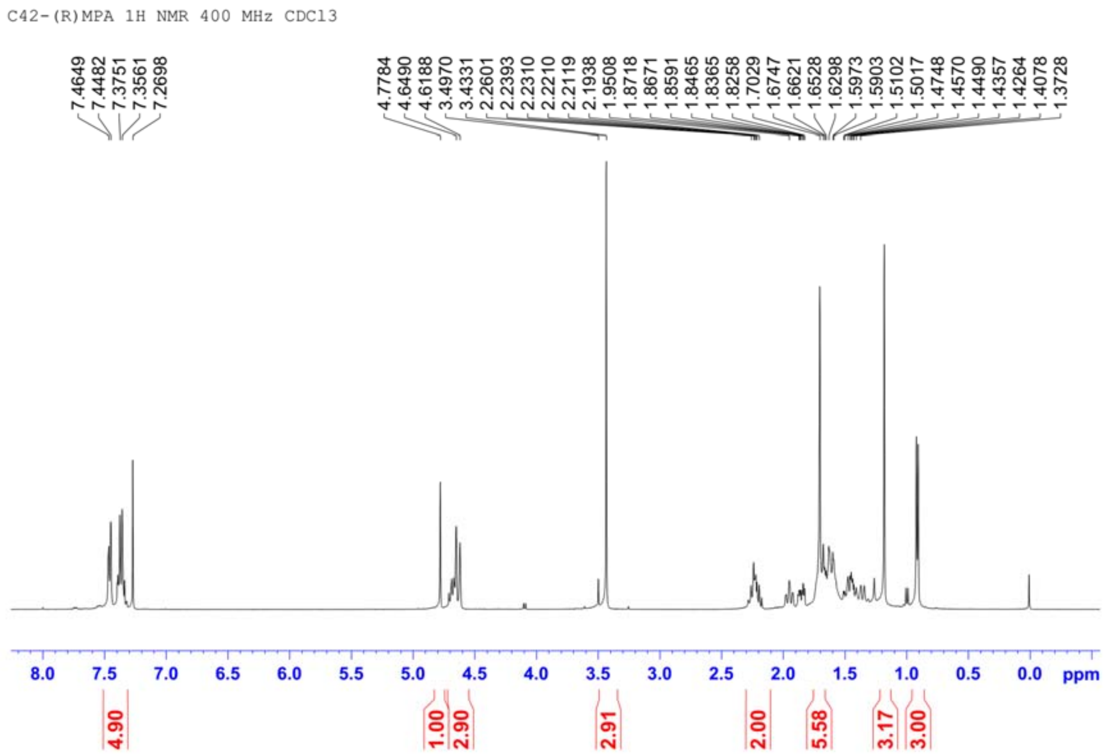

Figure S3-3. ¹H NMR spectrum of *R*-MPA ester of **3** (**3a**) in $CDCl_3$ (400 MHz).

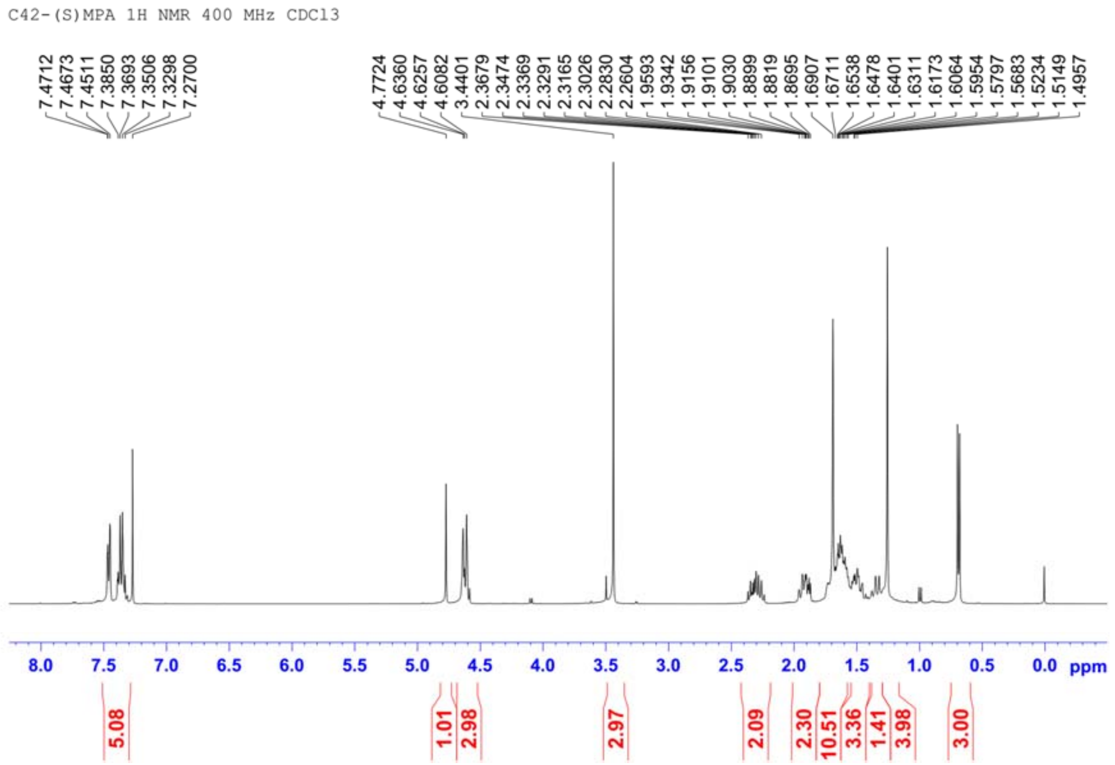

Figure S3-4. ¹H NMR spectrum of *S*-MPA ester of **3** (**3b**) in $CDCl_3$ (400 MHz).

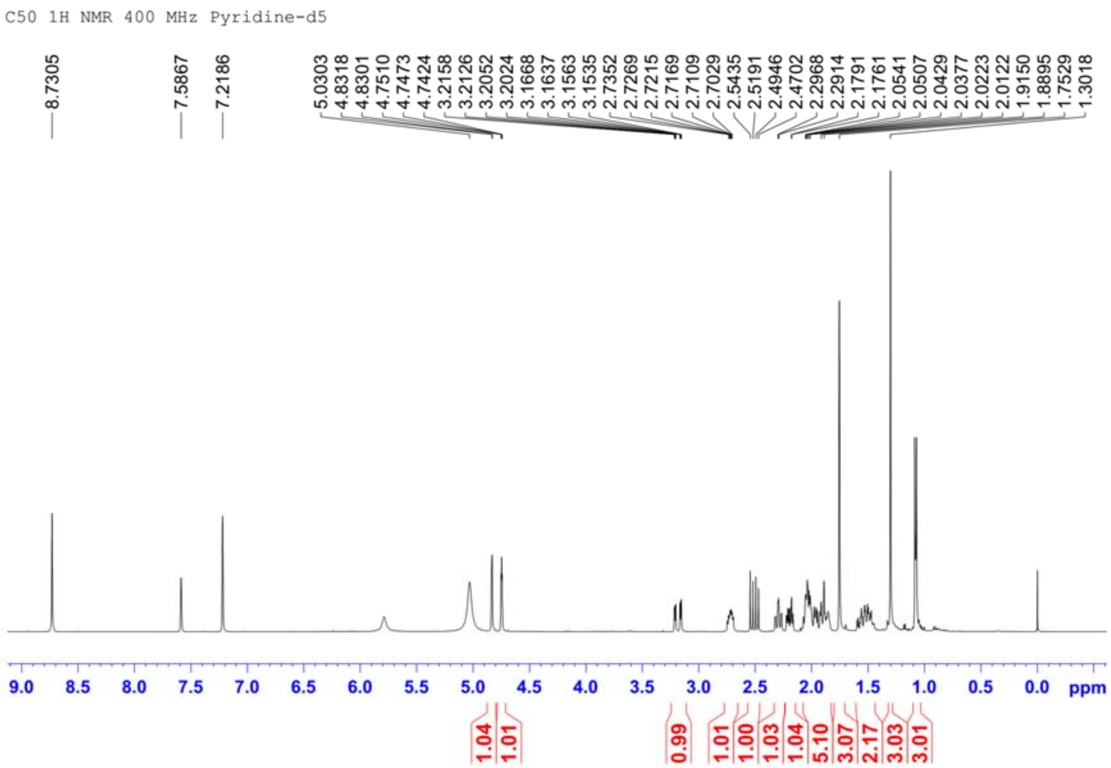

Figure S4-1. ^{1}H NMR spectrum of **4** in pyridine-d_5 (400 MHz).

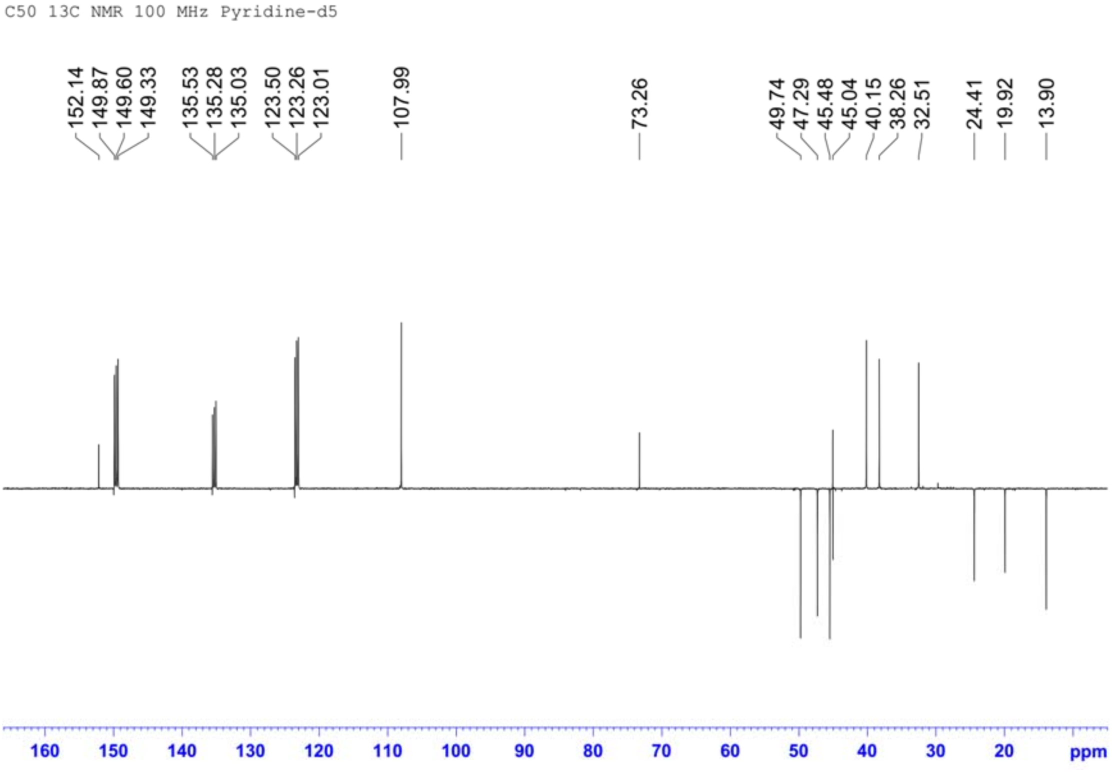

Figure S4-2. ^{13}C NMR spectrum of **4** in pyridine-d_5 (100 MHz).

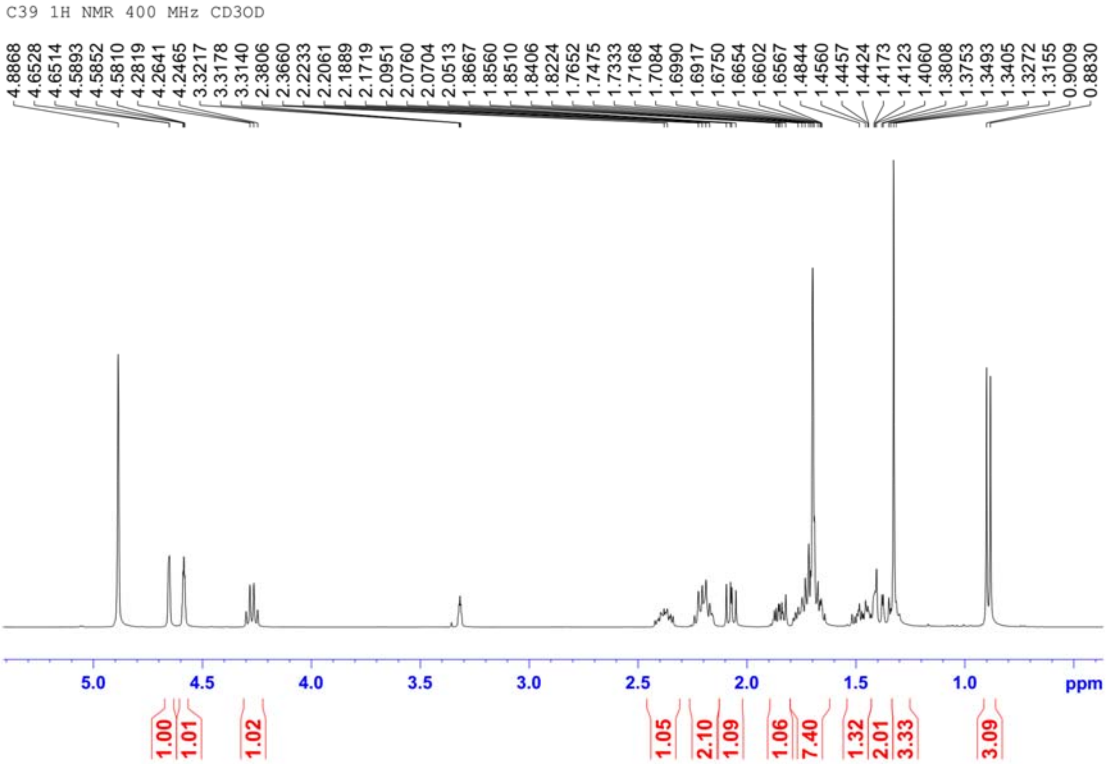

Figure S5-1. ^{1}H NMR spectrum of **5** in CD3OD (400 MHz).

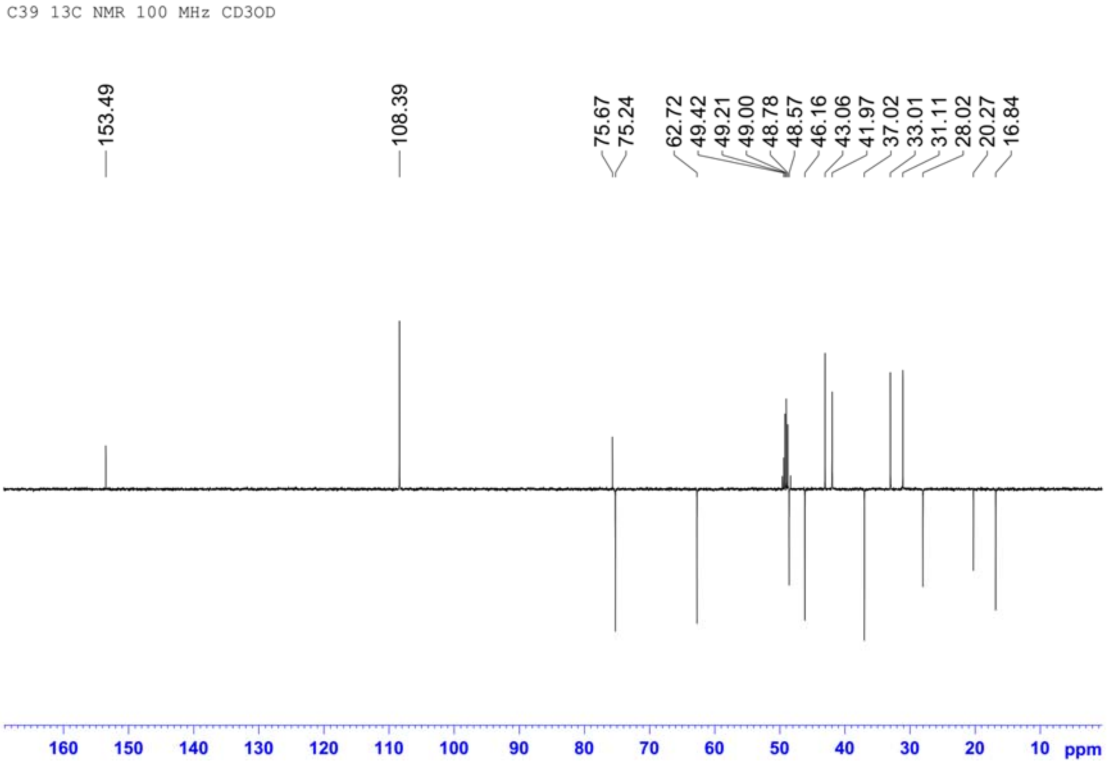

Figure S5-2. ^{13}C NMR spectrum of **5** in CD3OD (100 MHz).

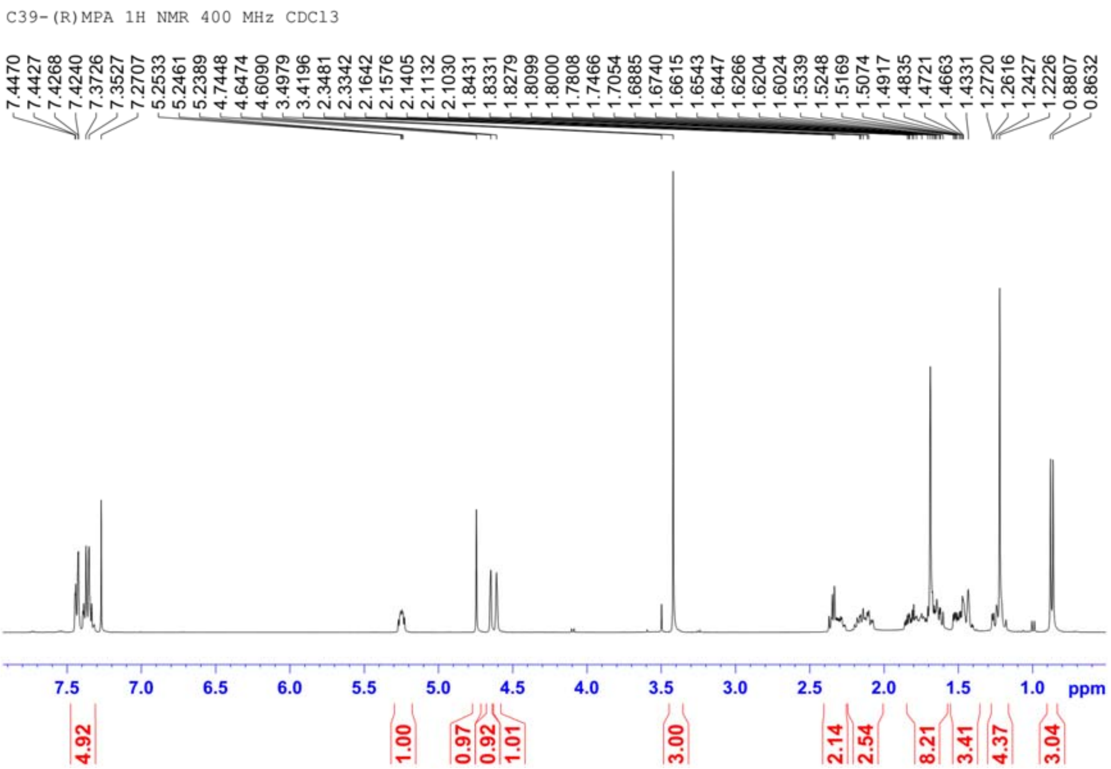

Figure S5-3. ^{1}H NMR spectrum of *R*-MPA ester of **5** (**5a**) in $CDCl_3$ (400 MHz).

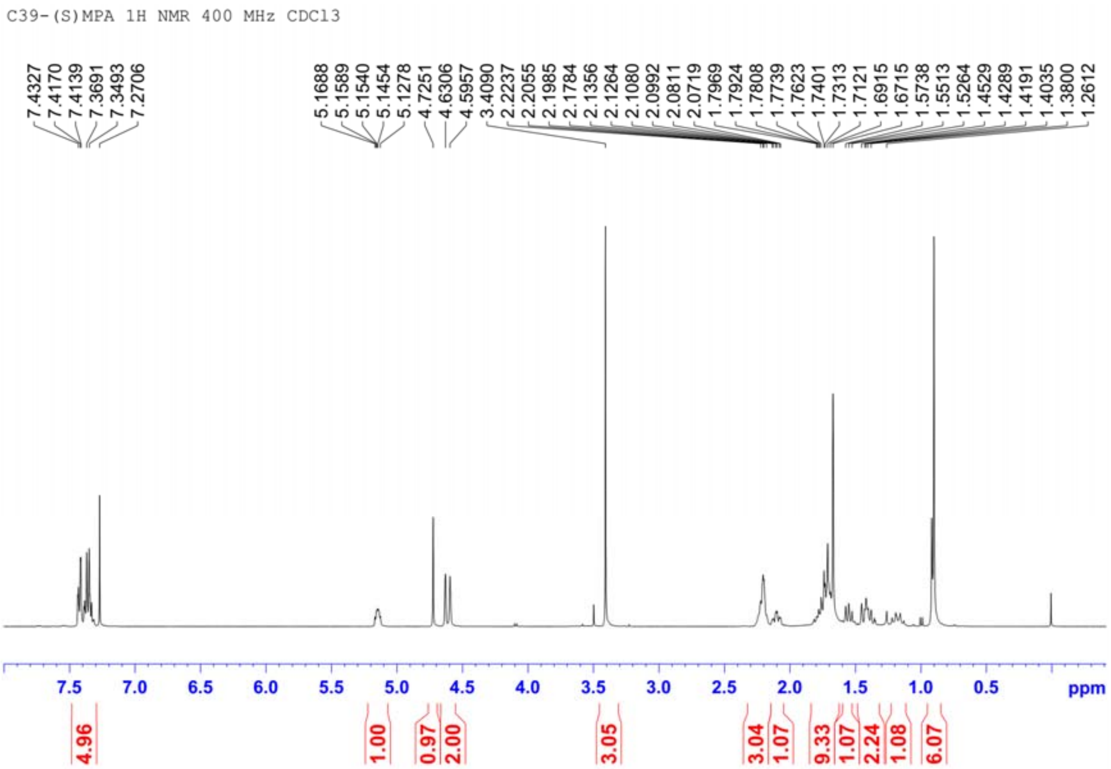

Figure S5-4. ^{1}H NMR spectrum of *S*-MPA ester of **5** (**5b**) in $CDCl_3$ (400 MHz).

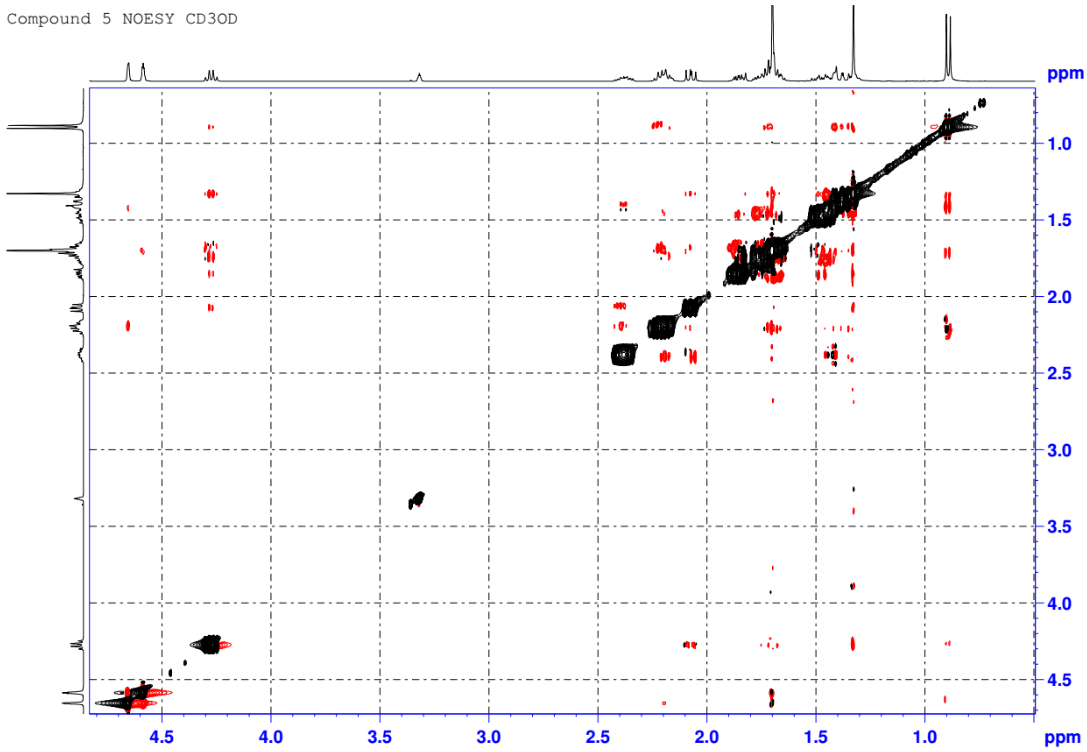

Figure S5-5. NOESY spectrum of **5** in CD_3OD (400 MHz).

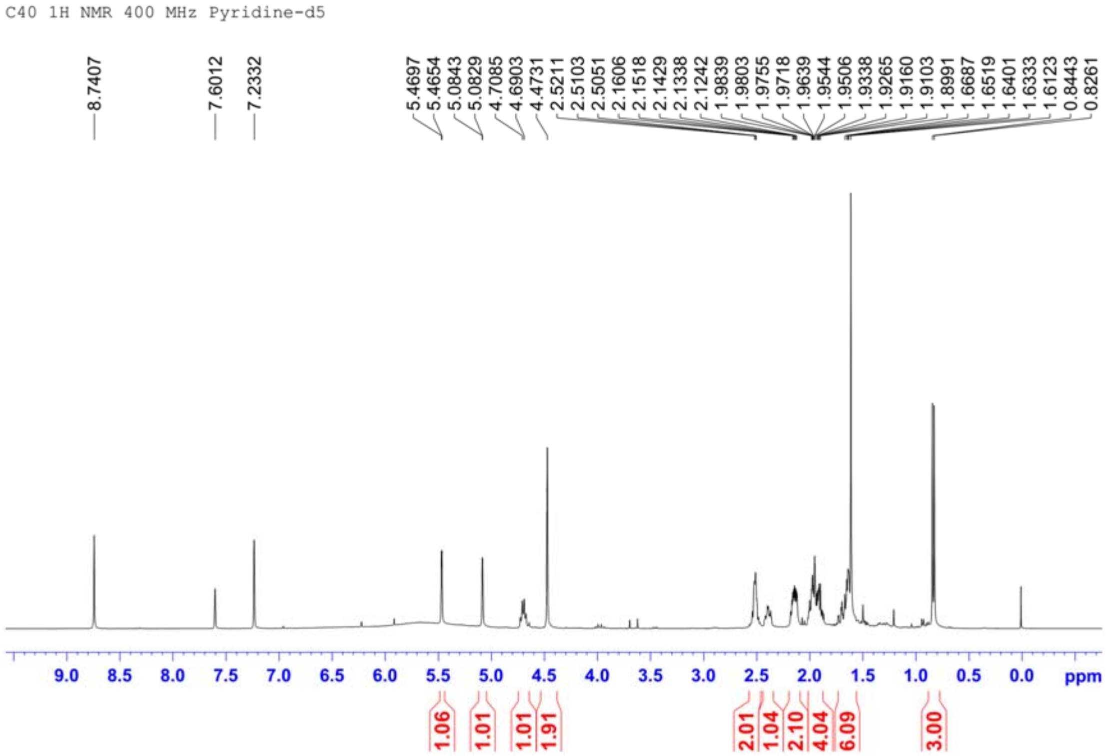

Figure S6-1. ^{1}H NMR spectrum of **6** in pyridine-d_5 (400 MHz).

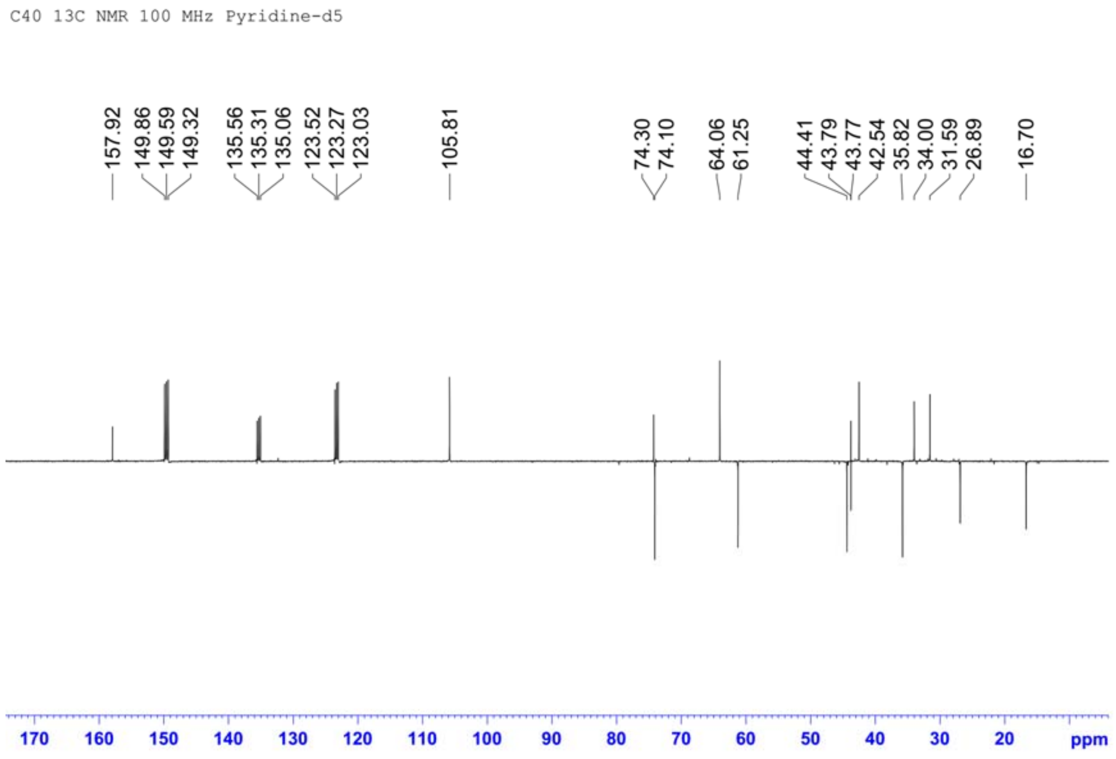

Figure S6-2. ^{13}C NMR spectrum of **6** in pyridine-d_5 (100 MHz).

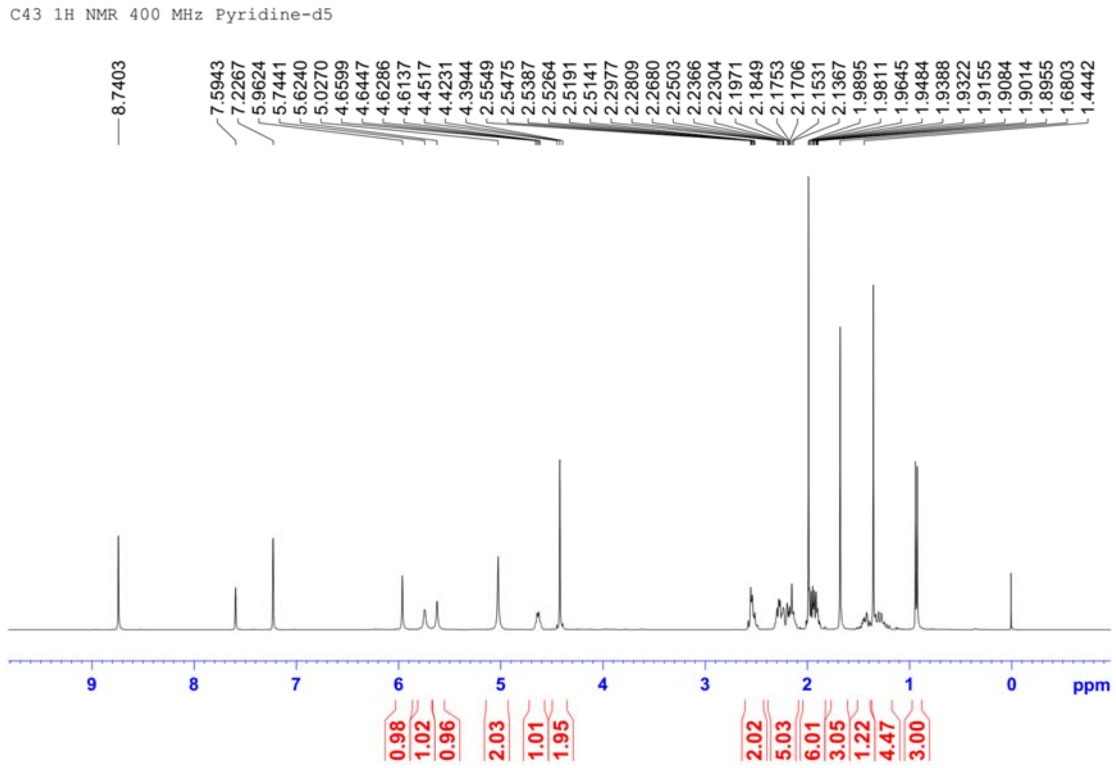

Figure S7-1. ^{1}H NMR spectrum of **7** in pyridine-d_5 (400 MHz).

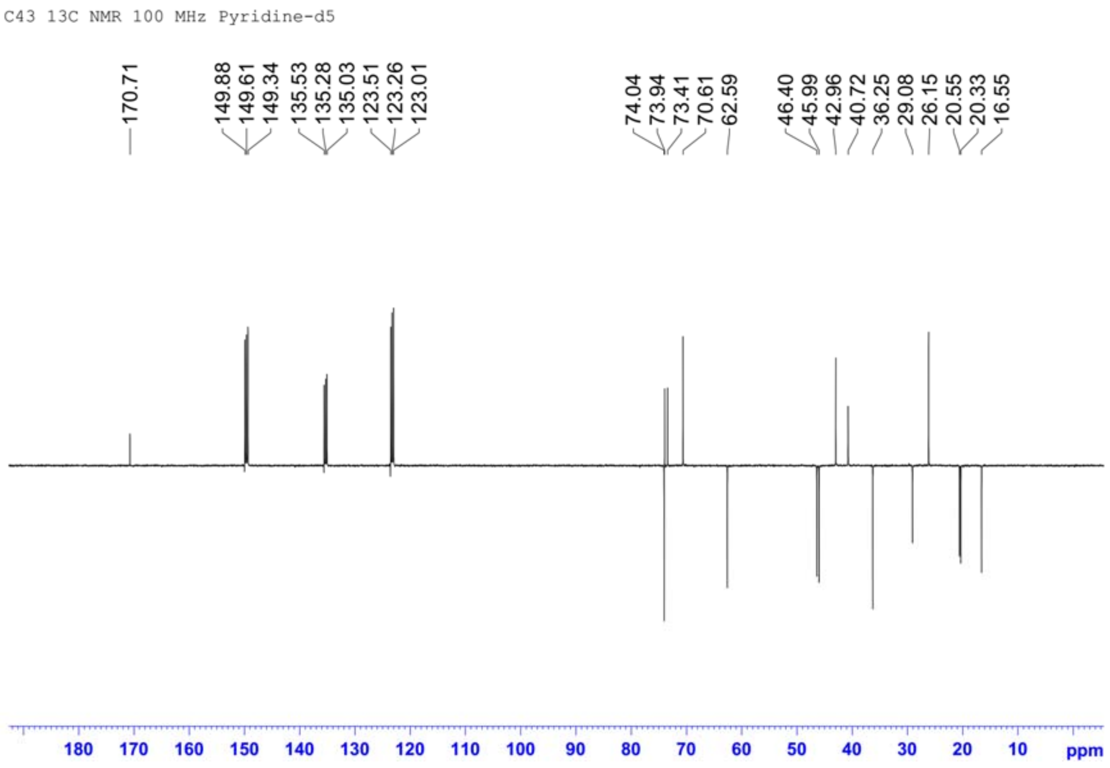

Figure S7-2. ^{13}C NMR spectrum of **7** in pyridine-d_5 (100 MHz).

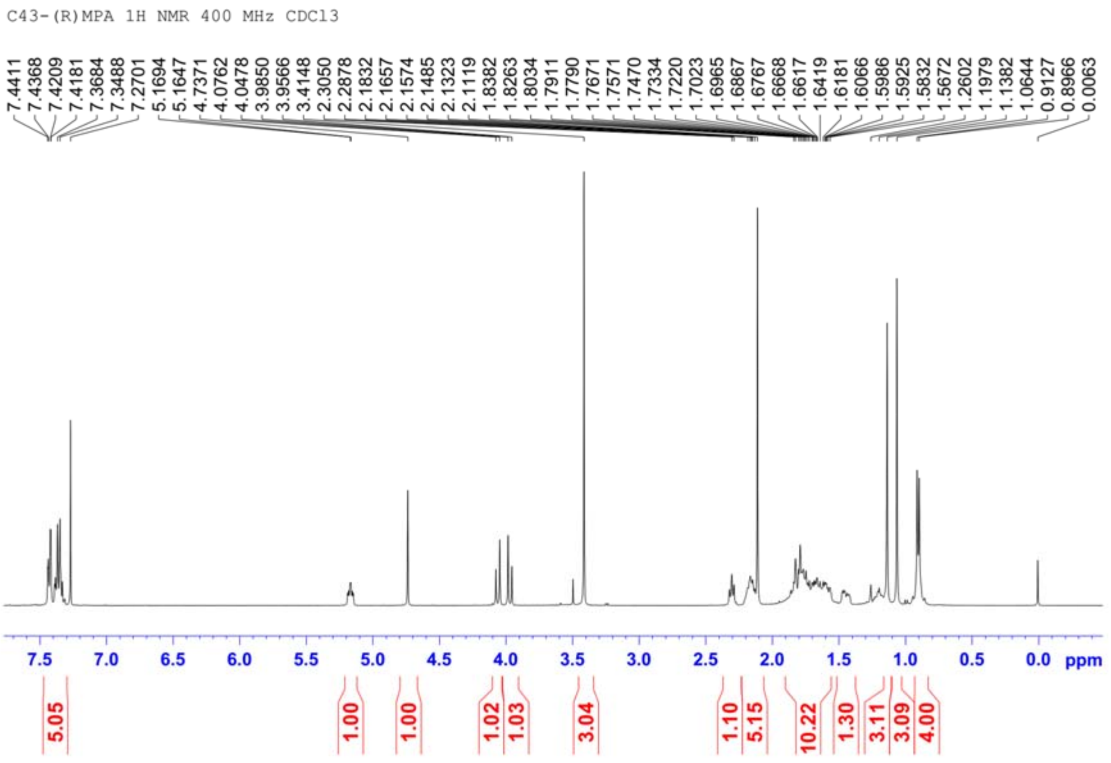

Figure S7-3. ^{1}H NMR spectrum of *R*-MPA ester of **7** (**7a**) in $CDCl_3$ (400 MHz).

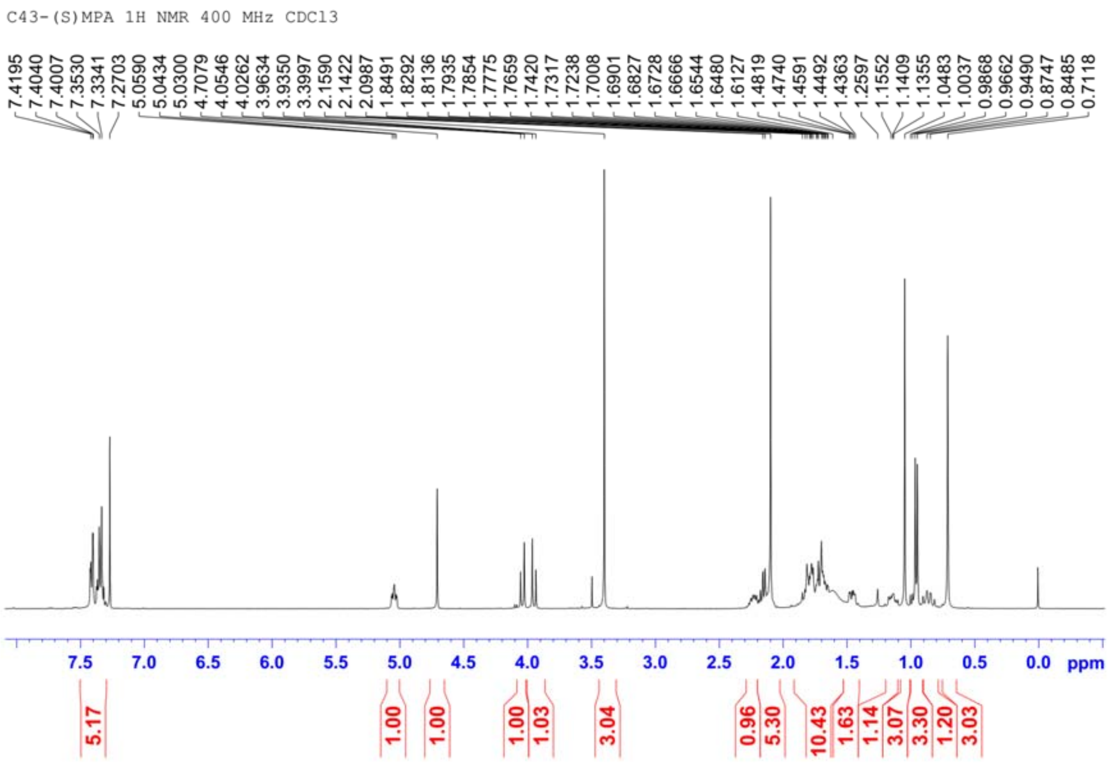

Figure S7-4.^{1}H NMR spectrum of *S*-MPA ester of **7** (**7b**) in $CDCl_3$ ((400 MHz).

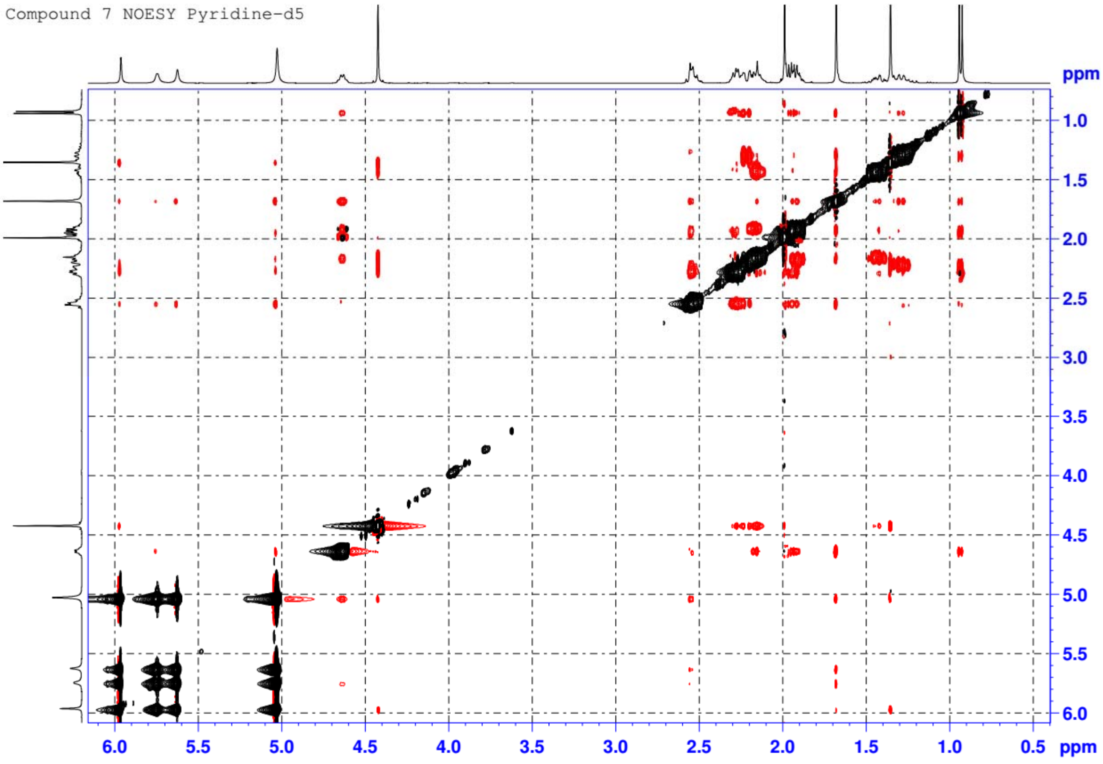

Figure S7-5. NOESY spectrum of **7** in pyridine-d_5 (400 MHz).

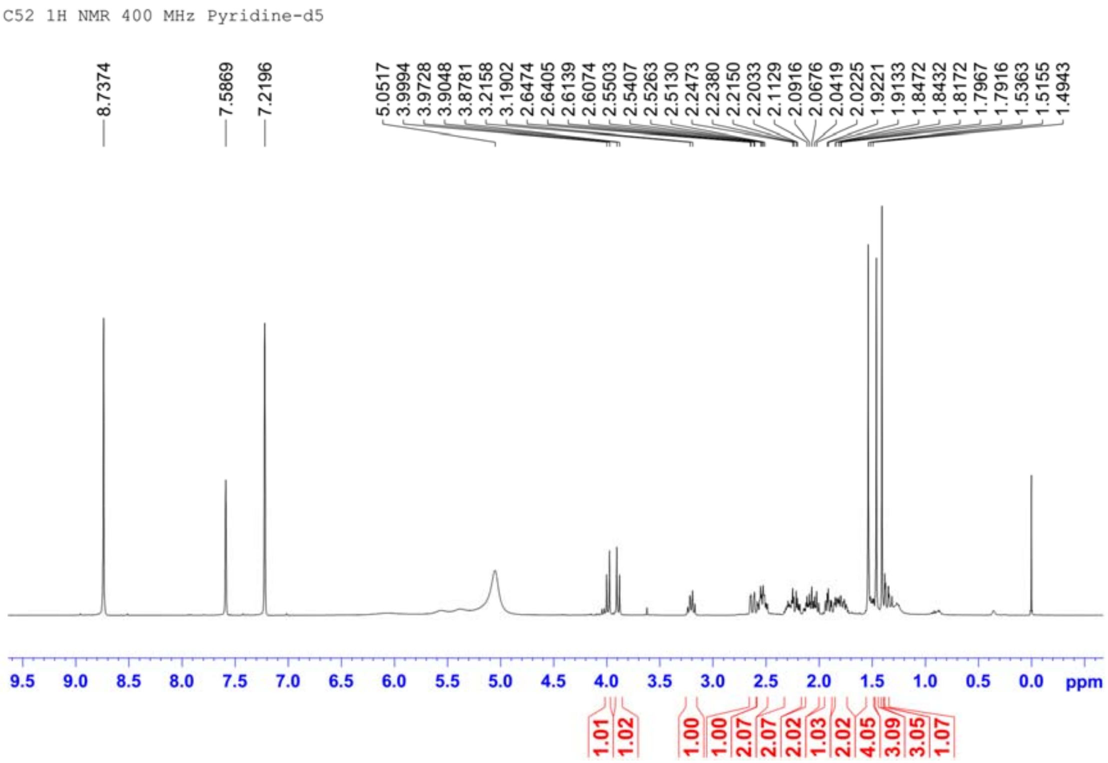

Figure S8-1. ^{1}H NMR spectrum of **8** in pyridine-*d*5 (400 MHz).

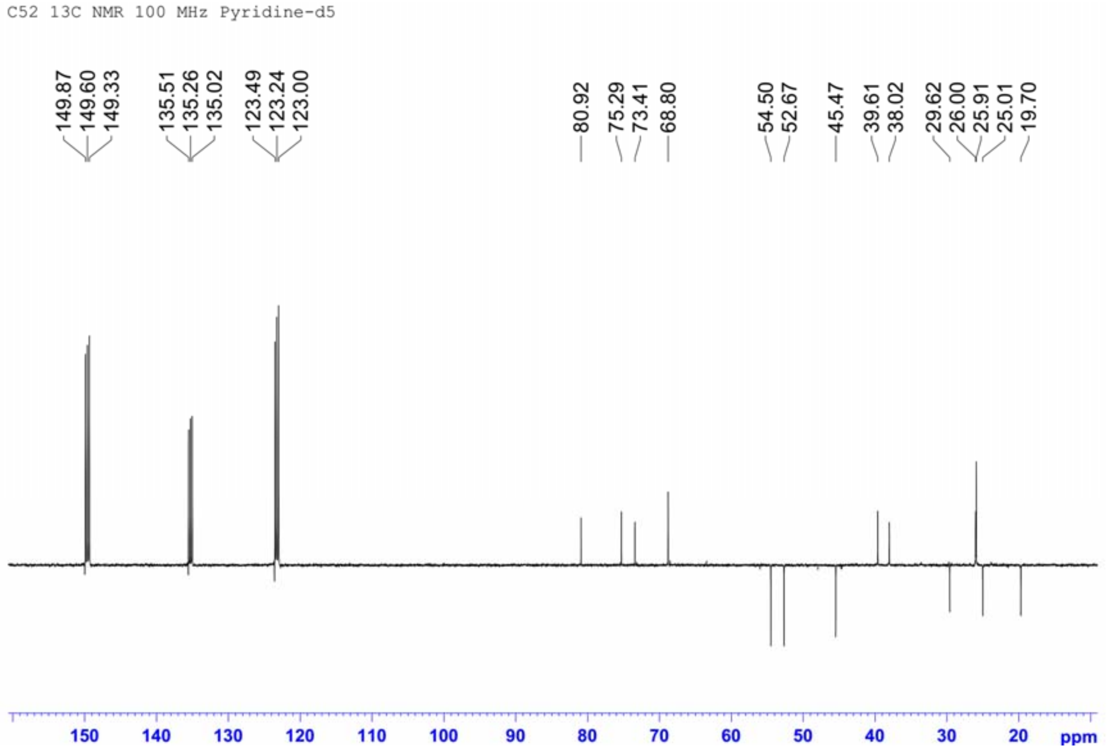

Figure S8-2. ^{13}C NMR spectrum of **8** in pyridine-*d*5 (100 MHz).

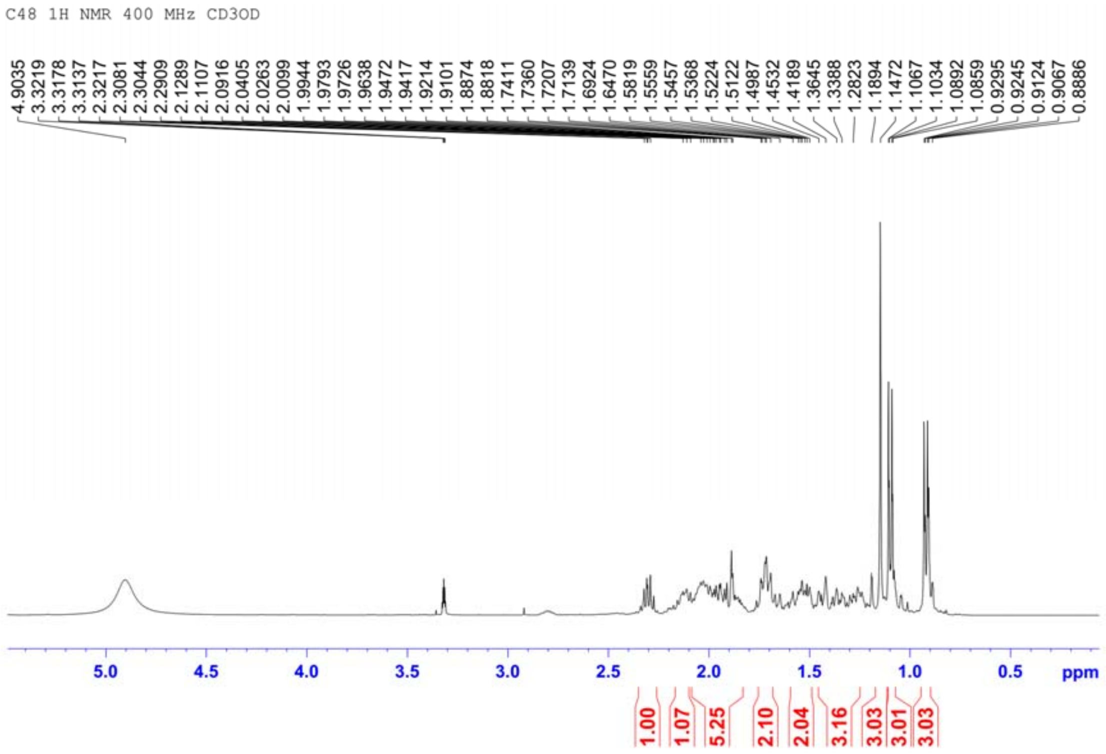

Figure S9-1. ^{1}H NMR spectrum of **9** in CD_3OD (400 MHz).

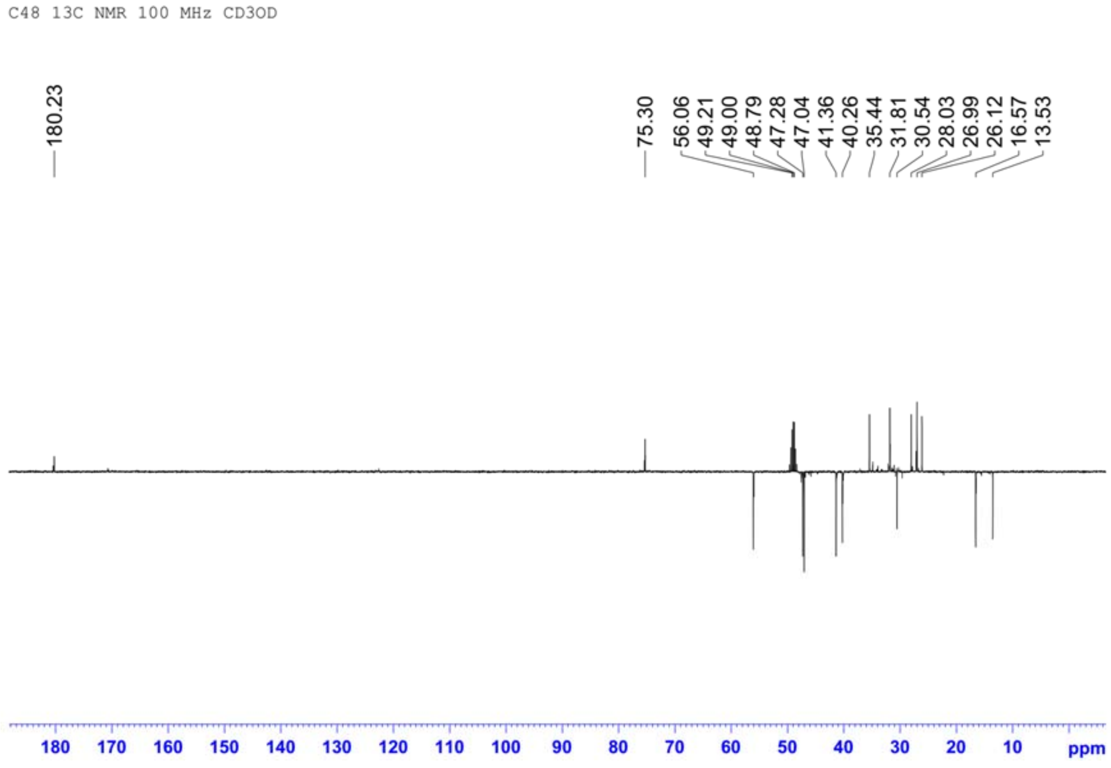

Figure S9-2. ^{13}C NMR spectrum of **9** in CD_3OD (100 MHz).
